# Aggregicyclins Shed Light on Type II Polyketide Biosynthesis in *Myxococcota*

**DOI:** 10.1021/jacsau.6c00256

**Published:** 2026-04-29

**Authors:** Chantal D. Bader, Sophia Panter, Fabian Panter, Amay Ajaykumar Agrawal, Olga V. Kalinina, Rolf Müller

**Affiliations:** a Department of Microbial Natural Products, Helmholtz-Institute for Pharmaceutical Research Saarland (HIPS), Helmholtz Centre for Infection Research (HZI) and Department of Pharmacy, Saarland University, Campus E8 1, Saarbrücken 66123, Germany; b Research Group Drug Bioinformatics, Helmholtz Centre for Infection Research (HZI), 443745 Helmholtz Institute for Pharmaceutical Research Saarland (HIPS), Saarbrücken 66123, Germany; c PharmaScienceHub, Saarbrücken 66123,Germany; d German Centre for Infection Research (DZIF), Partner Site Hannover-Braunschweig,Braunschweig 38124, Germany; e Center for Bioinformatics, Saarland University, Saarbrücken 66123,Germany; f Medical Faculty, Saarland University, Homburg 66421,Germany

**Keywords:** myxobacteria, specialized metabolites, biosynthesis, polyketide synthase, heterologous expression

## Abstract

Polycyclic bacterial specialized metabolites such as anthraquinones, angucyclines, and tetracyclines are predominantly produced by type II polyketide synthase (PKS) systems and represent an important source of antibiotics and anticancer agents. While type II PKS pathways are well characterized in Gram-positive bacteria, their biosynthetic potential in Gram-negative bacteria remains largely unexplored. Here, we report the discovery and activation of a cryptic type II PKS biosynthetic gene cluster from the myxobacterium *Aggregicoccus edonensis* MCy10622. Using a rapid PCR-based cloning and promoter-exchange strategy, the compact gene cluster was heterologously expressed in *Myxococcus xanthus* DK1622, leading to the production of two previously unknown specialized metabolites, aggregicyclin and oxyaggregicyclin. Structural analysis revealed an unusual polycyclic scaffold featuring a wide-spanning biaryl ether linkage. Biosynthetic analysis supports an early diversification event during first-ring cyclization as the basis for product divergence. Aggregicyclin exhibits antibacterial activity against *Staphylococcus aureus* and cytotoxicity against human cancer cells. Together, these findings expand the current knowledge of type II polyketide biosynthesis in Gram-negative bacteria.

## Graphical abstract

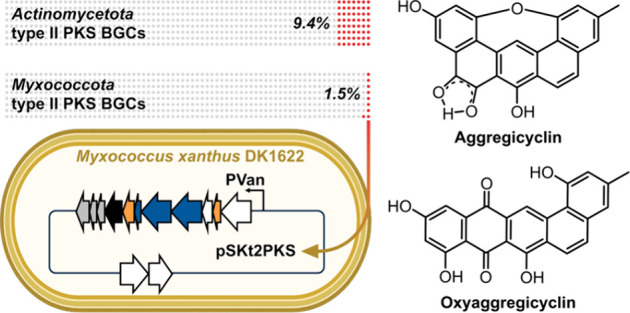

## 1 Introduction

Natural products (NPs)—in particular those of bacterial origin—have long been central to the discovery of new therapeutics.^1^ Anti-infective research and oncology have benefited especially from natural product-inspired molecules: most blockbuster antibiotics and many clinically important anticancer agents are directly or indirectly derived from NP scaffolds.^2^ Among the distinct NP families, type II polyketides stand out for their potent inhibitory activities against both prokaryotic and eukaryotic cells.^3^ This impact is exemplified by the tetracyclines, which account for an estimated 10–12% of the global antimicrobial market,^4^ and by the anthracycline doxorubicin, whose anticancer formulations had a global market value of over USD 1 billion in 2023.^5^ Notably, both of these cornerstone drugs were discovered from *Streptomyces* species during the mid-20th century, illustrating the enduring biomedical relevance of bacterial specialized metabolism.^6,7^

Type II polyketides are assembled by type II polyketide synthase pathways that are biosynthetically distinct from modular type I polyketide synthase (PKS) or nonribosomal peptide synthetases (NRPS), relying instead on iteratively acting enzymes. With the advent of the genomics era, genome mining has greatly expanded our understanding of how type II polyketides are biosynthesized.^3,8^ A distinct, iteratively acting PKS machinery assembles a linear poly-β-ketone chain, which is then cyclized and diversified by dedicated tailoring enzymes. Structural complexity typically emerges through late-stage oxidation, methylation, glycosylation, or other modifications introduced by cytochrome P450 monooxygenases, methyltransferases, and glycosyltransferases, among others.^9^ In contrast to modular BGCs, core genes in type II PKS biosynthetic gene clusters (BGCs) are comparatively compact.^10^ This compactness, together with a high degree of variation in tailoring enzymes, complicates the accurate prediction of cluster boundaries and often obscures the prediction of the final natural product when no chemical data are available.

Historically, nearly all characterized type II polyketides have been isolated from *Actinomycetota*, particularly from the genus *Streptomyces*, which has yielded paradigmatic scaffolds such as actinorhodin, tetracycline, doxorubicin, daunorubicin, oxytetracycline, numerous anthracyclines, angucyclines, and related families.^3^ Due to high reisolation rates within taxonomically similar strains, discovery efforts have increasingly turned toward underexplored bacterial phyla.^10^ Among these, the phylum *Myxococcota* has attracted substantial interest because of its remarkable, yet largely untapped, biosynthetic capability and chemical diversity. To date, however, only two type II polyketide families—the anthraquinone pyxidicyclins and the quinolone alkaloids aurachins—have been characterized from *Myxococcota*.^11,12^

In this study, we systematically surveyed type II polyketide biosynthetic potential across *Myxococcota* and identified a previously uncharacterized type II PKS BGC in *Aggregicoccus edonensis* MCy10622, a rare myxobacterium of the *Cystobacterinae* suborder.^13^ Because the pathway appeared to be cryptic in the native producer, we transferred the BGC into *Myxococcus xanthus* DK1622 and activated it through promoter exchange. This approach yielded two previously unknown type II polyketides, which we named aggregicyclins. Here, we describe their discovery, structural elucidation, and biological activities and propose a biosynthetic model for their formation based on gene annotation and chemical architecture.

## 2 Material and Methods

### 2.1 In Silico Analyses

BGCs were identified and annotated using antiSMASH version 7.0.^14^ For comparative analyses, BGCs belonging to *Myxococcota* and *Actinomycetota* from the public domain were retrieved from the antiSMASH database (version 4.0) and supplemented by in-house available *Myxococcota* genome sequences.^15,16^ To assess relatedness and diversity across genomes, BGCs were clustered into gene cluster families (GCFs) using BiG-SCAPE with a threshold of 0.5.^17^

Gene content, operon organization, and predicted enzymatic functions of *acy*BGC were analyzed manually to guide pathway activation strategies. Sequence homology analyses were performed with BLAST to assign putative functions to individual biosynthetic enzymes and to infer cyclization and tailoring reactions underlying specialized metabolite formations.^18^ Comparative analysis of the *acy*BGC with characterized type II PKS pathways was used to support biosynthetic hypotheses and to rationalize the structural features of the identified metabolites (see the Supporting Information for details). The sequence similarity network (SSN) was generated with EFI-EST using an alignment score of 33.^19^

### 2.2 Cloning and Heterologous Expression of the *acy*BGC

All oligonucleotide primers used for plasmid construction and verification are given in Table S6. PCR amplifications were carried out using Phusion high-fidelity DNA polymerase under standardized cycling conditions (Table S7). The pSKt2PKS expression vector was generated by stepwise modification of a p15A-based plasmid backbone, including replacement of the original coding region with a vanillate-inducible promoter–repressor system, insertion of the Mx8 prophage integrase for site-specific chromosomal integration, and incorporation of the two operons constituting the aggregicyclin BGC. The first operon was introduced by restriction–ligation cloning, whereas the second operon was integrated by λ-red-mediated recombineering in *Escherichia coli* GB08-red using overlap extension PCR products (Figures S3–S9). Final constructs were verified by restriction analysis and Illumina sequencing prior to transfer into *M. xanthus* DK1622 by electroporation. Stable single-crossover integration into the chromosomal Mx8 attB site was confirmed by diagnostic PCR. Detailed experimental procedures are described in the Supporting Information.

### 2.3 Analytical Scale Extraction, Analysis, and Data Processing

Growth media and cultivation conditions are listed in Tables S1–S3. Analytical-scale extraction of specialized metabolites was performed from frozen cell pellets by using a sequential acetone extraction protocol, followed by solvent evaporation and reconstitution in methanol prior to analysis. Clarified extracts were analyzed by UHPLC-high-resolution mass spectrometry using a reversed-phase C18 column coupled to an electrospray ionization time-of-flight mass spectrometer under standardized chromatographic and source conditions.^20^ Metabolite profiling was carried out using full-scan MS with UV–Vis detection, and mass calibration was performed automatically before each run using sodium formate clusters and internal lock masses. To identify metabolites specifically associated with heterologous expression of the aggregicyclin BGC, LC–MS data sets from wild-type *M. xanthus* DK1622 and the corresponding expression strain were compared using an unbiased principal component analysis-based workflow implemented in MetaboScape 5.0 (Bruker). Molecular features were detected using the T-ReX-3D algorithm and filtered based on reproducible presence in the expression strain and absence in the wild type. Targeted high-resolution tandem MS data were subsequently acquired by using scheduled precursor lists under collision-induced dissociation conditions. Detailed protocols and acquisition parameters are provided in the Supporting Information.

### 2.4 Large-Scale Production and Purification of Aggregicyclins

Production of the aggregicyclins was performed in six 5 L shake flasks filled with 2 L CTT medium each (recipe, see the Supporting Information) supplemented with 2% sterilized aqueous Amberlite XAD-16N suspension (50/50 w/v). After fermentation, cells and XAD-16N resin were harvested by centrifugation and lyophilized in the absence of UV light. The compounds were extracted using a 3:1 THF/MeOH mixture of THF/MeOH. THF was removed by evaporation under reduced pressure in the dark, and the extract was partitioned between MeOH and hexane. The methanolic layer was subsequently dried and again partitioned between chloroform and water, which led to the precipitation of a blackish-green sludge with a very high aggregicyclin content. This sludge was filtered off and dissolved in methanol. Two subsequent semipreparative HPLC steps resulted in pure aggregigyclin and oxyaggregicyclin (details, see the Supporting Information).

### 2.5 NMR-Based Structure Elucidation

1D and 2D NMR data was acquired in tetrahydrofuran-*d*_8_ on a Bruker Ascend 700 spectrometer equipped with a 5 mm TCI cryoprobe (^1^H at 700 MHz and ^13^C at 175 MHz). All observed chemical shift values (δ) are given in in ppm and coupling constant values (*J*) in Hz. Standard pulse programs were used for the HMBC, HSQC, and gCOSY experiments. HMBC experiments were optimized for^2,3^ *J*_C–H_ = 6, 8, and 10 Hz. The spectra were recorded in tetrahydrofuran-*d*_8_, and chemical shifts of the solvent signals at δH 1.73 ppm and δC 25.4 ppm were used as reference signals for spectra calibration.

### 2.6 Determination of Inhibitory Activities

Human HCT-116 colon carcinoma cells (ACC-581) were obtained from the German Collection of Microorganisms and Cell Cultures (DSMZ) and cultured under the conditions recommended by the depositor. For cytotoxicity assays, cells from exponentially growing cultures were harvested and seeded at 5 × 10^4^ cells per well in CELLBind 96-well plates containing 120 μL of modified McCoy’s 5A medium supplemented with 10% heat-inactivated fetal bovine serum (FBS). After a 2 h equilibration period, cells were treated with serial dilutions of the test compounds. Following 5 days of incubation at 37 °C, 20 μL of a 5 mg/mL thiazolyl blue tetrazolium bromide (MTT) solution in PBS was added. The medium was then discarded, and formazan crystals were dissolved in 100 μL of a 2-propanol/10 N HCl mixture (250:1). Absorbance was measured at 570 nm by using a Bio-Tek EL808 microplate reader.

All microorganisms used for biological assays were obtained from DSMZ or from an in-house strain collection and were cultured according to the depositor’s recommendations. Bacterial strains were grown in MHB (2.9 g/L beef infusion solids, 17.5 g/L casein hydrolysate, 1.5 g/L starch, pH 7.4), M7H9 medium (composition as specified, pH 6.6), or MYC 2.0 medium inoculated from agar-grown cultures. Test compounds were serially diluted in sterile 96-well plates prior to the addition of the bacterial cell suspension. Cultures were incubated for 24 h at room temperature, 30 °C, or 37 °C, depending on the strain. Growth inhibition was assessed visually, and MIC_5_0 values were calculated relative to untreated controls using sigmoidal curve fitting. Positive controls for each microbial test strain are listed in Table S19.

## 3 Results and Discussion

### 3.1 Type II PKS Are Rare in *Myxococcota*

To gain deeper insight into the specific biosynthetic features distinguishing *Myxococcota* from *Actinomycetota*, we examined the distribution and diversity of the key biosynthetic classes. Interestingly, while PKSs of all types are similarly abundant in *Myxococcota* and *Actinomycetota* (∼11.8% in *Myxococcota* vs ∼ 15.6% in Actinomycetota), type II PKS systems are relatively rare in *Myxococcota* (1.5 vs 9.4% in *Actinomycetota*, Figure 1A). Using a genome-mining approach, we systematically retrieved all type II PKS BGCs from *Myxococcota* genomes and clustered them together with experimentally characterized BGCs to identify GCFs.^15–17,21^ The majority of type II PKS BGC group into singleton GCFs, showing no similarity to already described BGCs and indicating their products to be chemically unique. One exception is one GCF containing three BGCs from two *Myxococcota* genera, but again no experimentally described BGCs (Figure 1B). To explore this conserved GCF, we performed a more detailed in silico analysis of the *acy*BGC from *A. edonensis* MCy10622 and *Melittangium boletus* Meb2 (Figures S1 and S2): the *acy*BGC is rather short consisting solely of two operons with a total length of 9691 kbp with 13 CDS called *acyA* to *acyM* (Figure 1C). The first operon consists of the CDS *acyA* to *acyK,* the second one of *acyL* to *acyM*. The surrounding regions around these two operons are unlikely to be involved in biosynthesis of the PKS type II product, as they do not encode enzymes known to be involved in bacterial specialized metabolism. No ABC-type exporter system was found in or around the BGC, which indicates the PKS type II small-molecule product is either exported through passive mechanisms or utilizes a broader range exporter system encoded in a different locus on the bacterial genome (Tables S4 and S5).

**Figure 1:**
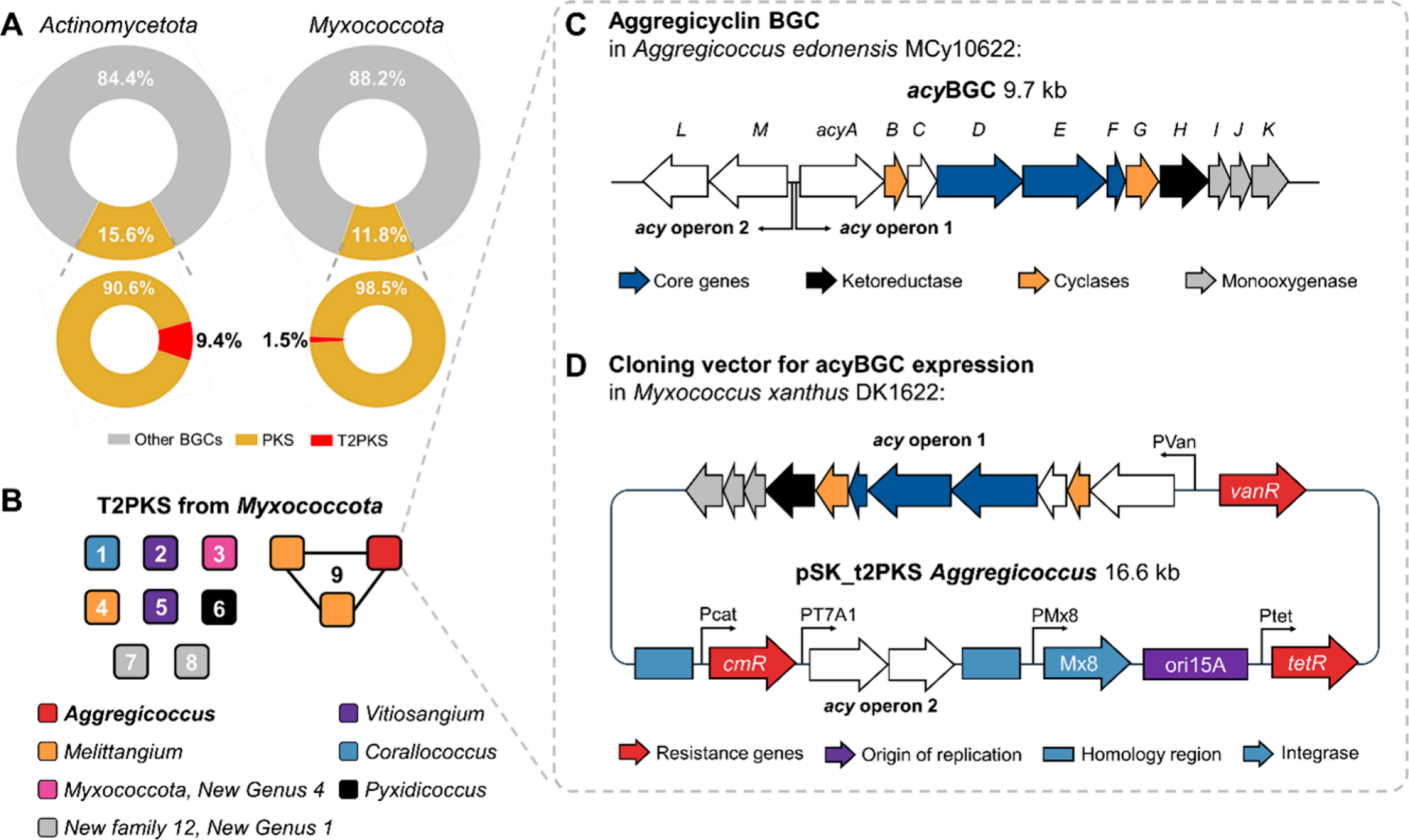
Survey of type II PKS in *Myxococcota* and cloning strategy of the aggregicyclin BGC to achieve the production of aggregicyclins. (A) Percentage of PKS and type II PKS in *Actinomycetota* vs *Myxococcota*. (B) Type II PKS BGCs in *Myxococcota* cluster into nine gene cluster families (GCFs). BGC 8 = *pcy*BGC.^11^ (C) In silico analysis of the cryptic aggregicyclin BGC (*acy*BGC) from GCF 9 reveals a two operon structure comprising three core genes (*acyD–F*), two cyclases (*acyB* and *acyG*), one ketoreductase (*acyH*), and three monooxygenases (*acyI-K*). (D) The two *acy*BGC operons were amplified separately and cloned into a p15A-based vector to put them under the control of exogenic promoters before transformation into *M. xanthus* DK1622 to eventually achieve production of the aggregicyclins.

Next, we aimed to assign a product to the *acy* BGC. As this BGC was cryptic in the native producer, heterologous expression in the model host *M. xanthus* DK1622 was chosen as the most suitable strategy to enable the production of the encoded specialized metabolites. Because the *acy*BGC is relatively small—reflecting the iterative nature of type II PKS systems, which do not rely on large megasynthase enzymes—it was not necessary to employ cloning approaches designed for large genomic fragments, such as exoCET (exonuclease combined with RecET recombination) or TAR (transformation-associated recombination), nor to use assembly strategies like Gibson or Golden Gate cloning.^22–24^

The first operon was rather amplified by PCR and subsequently cloned into a p15A-based cloning vector that already contained the vanillate promoter and repressor cassette by restriction cloning (Figure 1D and Figures S3–S6).^25^ This promoter was chosen for cluster activation as it has shown to lead to stringent expression control and strong expression of BGCs upon activation in several myxobacteria.^11,26,27^ Furthermore, the vector is equipped with an Mx8 prophage integrase that can transfer and integrate the vector DNA into the bacterial chromosome of the myxobacterial *attB* site to allow for stable and reliable expression of the cloned BGC.

The second operon was amplified by PCR and subsequently fused to a chloramphenicol acyl transferase (*cat* gene) as a selection marker and a tn5 promoter via overlap extension PCR (Figure S7). This PCR fragment was integrated into pSKt2PKS operon1 by λ-red-mediated recombineering in *E. coli* GB08-red (Figure S8).^28^ The obtained vector could then be used to transfer the *acy*BGC featuring exogenic promoters into our myxobacterial model strain *M. xanthus* DK1622 (Figure 1D and Figure S9).

### 3.2 Aggregicyclins Act as Autoinducers on the Vanillate Promotor

Analysis of the transformants’ methanolic extracts showed the appearance of peaks at *m*/*z* 371.09 [M + H]^+^ for the aggregicycline isomers and a peak at *m*/*z* 387.08 [M + H]^+^ for oxyaggregicycline in about 50% of the transformants that were verified to harbor the pSKt2PKS plasmid (Figure 2A and Figures S10–S13). It became obvious quickly that in comparison to other plasmids of the size of pSKt2PKS, transformation efficiency was very low and growth of the resulting *M. xanthus* DK1622::pSKt2PKS was significantly reduced. This could already be seen as an early hint on autotoxicity associated with expression of the pSKt2PKS plasmid.

**Figure 2:**
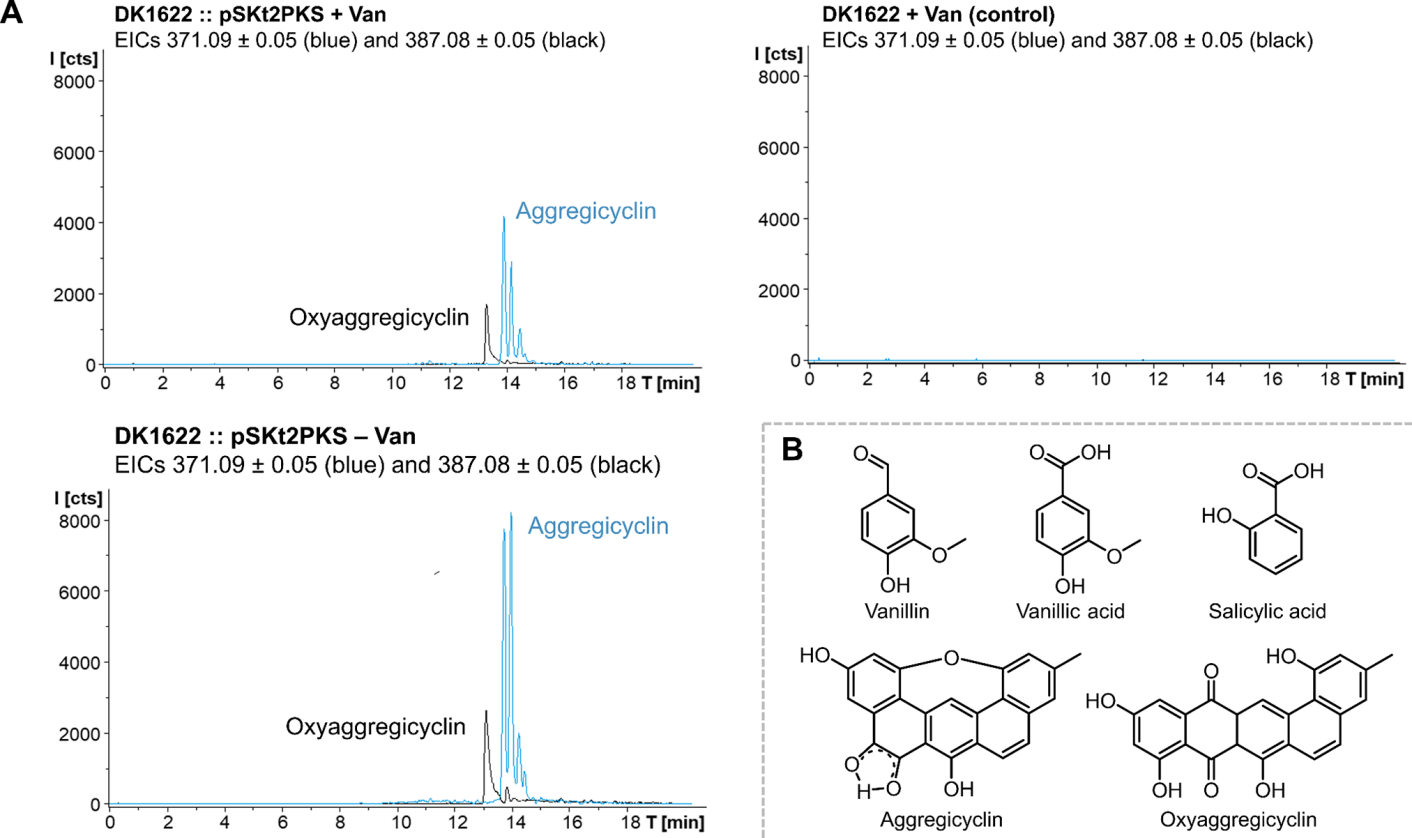
Expression of pSKt2PKS in *M. xanthus* DK1622 yields two polyketides with autoinduction of their production. (A) Extracted ion chromatograms (EICs) at *m*/*z* 371.09 for aggregicyclin and 387.08 for oxyaggregicyclin showing production in both the vanillate induced and noninduced heterologous production system that are absent in wild-type *M. xanthu*s DK1622. (B) Small aromatic compounds are known to induce the vanillate promoter in comparison with the isolated aggregicyclin isomers.

Interestingly, we observed that production of aggregicyclins in noninduced *M. xanthus* DK1622::pSKt2PKS transformants and supplement of vanillate did not increase aggregicycline production (Figure 2A), which could hint toward an autoinduction in our system. A possible explanation for this observation is that the vanillate promoter–repressor system, which is known to respond to a relatively broad range of lignin-derived compounds,^29^ is induced by bi- or degradation products from the aggregicyclin biosynthesis showing structural similarities to vanillate, vanillic acid, or salicylic acid (Figure 2B). This finding should be considered in future experiments when aiming to produce small aromatic compounds using vanillate promoter-based expression of the corresponding biosynthetic proteins.

### 3.3 Aggregicyclin and Oxyaggregicyclin Present Distinct Cyclization Patterns

Upon production of the aggregicyclins in preparative scale, we realized that both aggregicyclin as well as oxyaggregicyclin quickly degrade in both ambient light as well as the UV cell of the semipreparative HPLC system. In addition to that, basic conditions as well as strong acidic conditions also led to complete degradation of the aggregicyclines, which had to be considered during the workup process. To counteract this, both cultivation and compound purification by liquid/liquid separation followed by semipreparative HPLC were carried out in the dark at neutral pH. Structure elucidation of the two natural products was performed by 2D NMR analysis (Figure 3) with the final structure supported by biosynthetic logic of the proposed aggregicyclin biosynthetic pathway (see Section 3.4).

**Figure 3:**
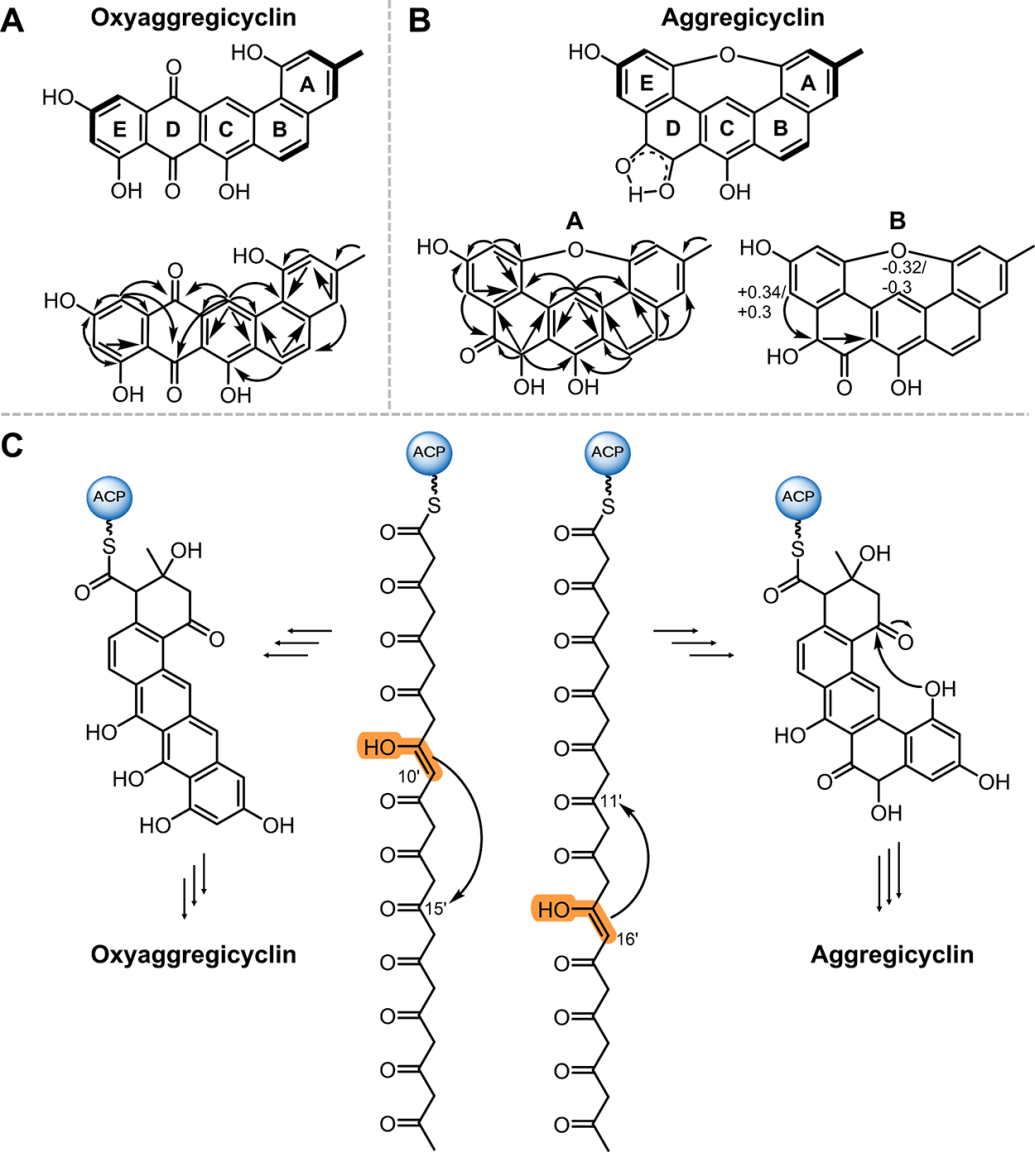
Two aggregicyclin derivatives present distinct cyclization patterns likely caused by a distinct first-ring cyclization. (A) Chemical structure of oxyaggregicyclin with most relevant COSY (bold) and HMBC (arrows) correlations used for structure elucidation. (B) Chemical structure of the two aggregicyclin isomers with the highest production titers (A and B) with the most relevant COSY (bold) and HMBC (arrows) correlations used for structure elucidation. Most affected chemical shifts between A and B marked in the panel (B) structure. (C) The divergent chemical structures of aggregicyclin and oxyaggregicyclin are plausibly explained by a promiscuity of the first cyclase AcyG acting at distinct positions. Details of the biosynthesis are given in Figure 4.

Due to the rapid isomerization of aggregicyclin, structure elucidation was first performed for oxyaggregicyclin (Figure 3A and Figures S14 and S15). The ^1^H NMR and HSQC spectra of oxyaggregicyclin revealed signals characteristic for seven aromatic methines at δH 10.27 (1H, s) δC 122.9, δH 8.34 (1H, d, *J* 9.0) δC 121.5, δH 7.85 (1H, d, *J* 9.0) δC 130.7, δH 7.32 (1H, s) δC 121.1, δH 7.28 (1H, s) δC 109.2, δH 7.00 (1H, s) δC 116.0, and δH 6.58 (1H, bd, *J* 2.2) δC 108.3 ppm and one signal characteristic for a methyl group substituting an aromatic ring at δH 2.49 (3H, s) δC 21.3 ppm (Table S8 and Figures S21–S26). Only two aromatic protons were found to show coupling constants greater than 3 Hz, indicating them as the only aromatic protons in the ortho position to each other.

All protons were assigned to substitute the five six-membered aromatic rings A–E (Figure 3A) based on their COSY and HMBC correlations, as well as the characteristic chemical shifts of the quaternary carbons serving as bridgehead atoms between the different rings (for a detailed description, see the Supporting Information). The substitution pattern of the hydroxy functions as well as the two keto functions was elucidated based on their characteristic deshielded chemical shifts and respective HMBC correlations with the aromatic protons. Based on the NMR data, two substitution patterns were possible in the E ring, as the chinone system in ring D interrupts all NMR correlations between protons participating in the C and E rings. Our investigation of the biosynthetic formation of oxyaggregicyclin—which is described in detail in the following Section 3.4—however allowed us to predict with confidence the substitution pattern depicted in Figure 3C. Notably, the core scaffold of oxyaggregicyclin closely resembles that of bequinostatin D, reported from *Streptomyces* sp. MI384-DF12. Both compounds share the same arrangement and substitution pattern across all five aromatic rings, with the only difference being the E ring, which bears a propyl substituent in bequinostatin D instead of a methyl group, as in oxyaggregicyclin. Consistent with this structural similarity, the reported proton and carbon chemical shifts of the shared moieties in bequinostatin D are in good agreement with those determined for oxyaggregicyclin.^30^

Once we had the oxyaggregicyclin structure in hand, we were able to determine the aggregicyclin structure (Figure S16), consisting of two isomers observable in the NMR spectra, which we called aggegricyclin A and B (Figure 3B). Additional to seven signals characteristic for aromatic protons and one signal characteristic for a methyl group substituting an aromatic ring, we detected a methine group at δH 4.74 (3H, s) δC 58.9 ppm missing in oxyaggregicyclin (Table S9 and Figures S27–S34).

The chemical shifts of signals in aggregicyclins A and B resemble those in oxyaggregicyclin, while chemical shifts in the D ring differ (Tables S8 and S9). In contrast to oxyaggregicyclin, we only detect three exchangeable protons at δH 12.92 (A)/12.70 (B), 12.00, and 7.93 ppm. We could not detect any correlations of the proton at 7.93 ppm, but this proton could be assigned based on its characteristic chemical shift as a secondary hydroxy function. This assignment is further supported by the finding that we detect four isomers in our UHPLC-MS analysis, which likely correspond to the keto–enol tautomers of aggregicyclin with the two possible stereoconfigurations of the secondary alcohol (Figure 2A and Figures S17 and S19).

The equilibrium is pH-dependent, which explains why the earlier-eluting peak corresponding to aggregicyclin B predominates in the crude extract at neutral pH, whereas the later-eluting peak corresponding to aggregicyclin A is more abundant in the NMR sample under slightly acidic conditions. Under acidic conditions, the keto–enol tautomerism also appears to proceed more rapidly, as reflected by the detection of only the enol form in the crude extract, but not in the NMR sample. Two HSQC signals are mainly affected by isomerization of aggregicyclin A to B (Figure 3B). The deshielding shift of the ketone in comparison to that of the hydroxy function is hereby clearly visible. In aggregicyclin B, we observe correlations of the aromatic proton assigned to the E to the methine carbon, which underpins our hypothesis regarding isomerization in this position. Together with the observed chemical shifts in aggregicyclin, its sum formula indicates a biarylether linkage in the molecule. Unfortunately, the proton at δH 12.03 ppm displays only weak correlations hardly above the signal-to-noise ratio, wherefore we were unable to pinpoint its precise location (Figures S32–S34). However, based on correlations observed in the 2D NMR spectra, we could unambiguously assign two fragments of the aggregicyclin structure as A and B and D and E rings (Figure S18, fragments 1 and 2). However, there are four possibilities of how these fragments could be connected by the remaining aromatic C ring (Figure S18, **1**–**4**).

Structures **3** and **4** were excluded based on the observed hydroxylation pattern in the E ring, which is contradictory to predicted positions stemming from the incorporation of acetate units during biosynthesis. Both structure **1** and **2** show the expected hydroxylation pattern, but the acquired NMR data strongly suggests **2** as the structure of aggregicyclin (Figure 3B). The hydroxy proton at δH 12.92 ppm thereby represented the decisive structural feature: it exhibits HMBC correlations to three quaternary carbons, of which one was assigned as hydroxy-bearing carbon based on its characteristic chemical shift of δC 195.9 ppm (Figure S18 and Table S9). The other two were assigned as the neighboring quaternary carbons based on their correlations to the protons in rings B and D, suggesting this proton to be carried by the hydroxy function in ring C. The positioning of a free hydroxy function in the C ring is additionally supported by the deshielded shift of the respective proton at δH 12.92 ppm, which better fits the predicted chemical shift of a proton in this position at δH 11.30 ppm in comparison to a free hydroxy function in the A ring δH 9.04 ppm and resembles the deshielding effect of neighboring ketones in aromatic polyketides as described for tiancimycin exemplarily.^31^ To underpin our proposed aggregicyclin connectivity by visualization of long-range HMBC correlations, we performed HMBC experiments with varying constant 13 values (6, 8, and 10 Hz) and changed the NMR solvent to deuterated methanol. Unfortunately, both attempts failed to provide the required information. CIGAR-HBMC experiments were also unsuccessful, most likely due to their comparably low sensitivity.^32^ Due to the very poor solubility of aggregicyclin in most chemical solvents, we were unable to acquire NMR data in additional solvents. Unfortunately, even though substantial efforts were undertaken, crystallization experiments were not successful, and due to the low production titers and fast degradation, we were unable to obtain sufficient amounts for solid-state NMR.

### 3.4 Aggregicyclin and Oxyaggregicyclin Likely Derive from an Early Biosynthetic Diversification Event

Having solved the chemical structure of the aggregicyclins, we were able to investigate their biosynthesis in more detail. As both derivatives stem from the same biosynthetic machinery, we postulate that structural divergence stems from a distinct first-ring cyclization (Figure 3C). To gain further insights, we aligned the biosynthetic steps necessary to produce the aggregicyclins with the gene functions in the heterologously expressed BGC (Tables S4 and S5).

Aggregicyclin biosynthesis starts, as expected in type II PKS biosynthesis, with a concatenation of malonyl-CoA building blocks to a polyketone (Figure 4A). The underlying reactions are performed by an enzyme complex consisting of a keto synthase (KS) α, a KSβ, and an acyl carrier protein (ACP). In this case, the complex concatenates 11 malonyl-CoA units with the starter moiety acetyl-CoA to form the precursor consisting of 24 carbon atoms forming the aggregicyclin backbone. This highly reactive polyketone precursor is cyclized in a first aldol condensation and aromatization step performed by the first-ring cyclase AcyG forming the A-ring. AcyG shows homology to TcmN, the first-ring cyclase involved in tetracenomycin biosynthesis, as well as BenH from the benastatin biosynthetic pathway (see Figure S20), which is well aligned with the C11′ to C16′ aldol condensation/aromatization reaction that is catalyzed during aggregicyclin formation.^33–35^

**Figure 4:**
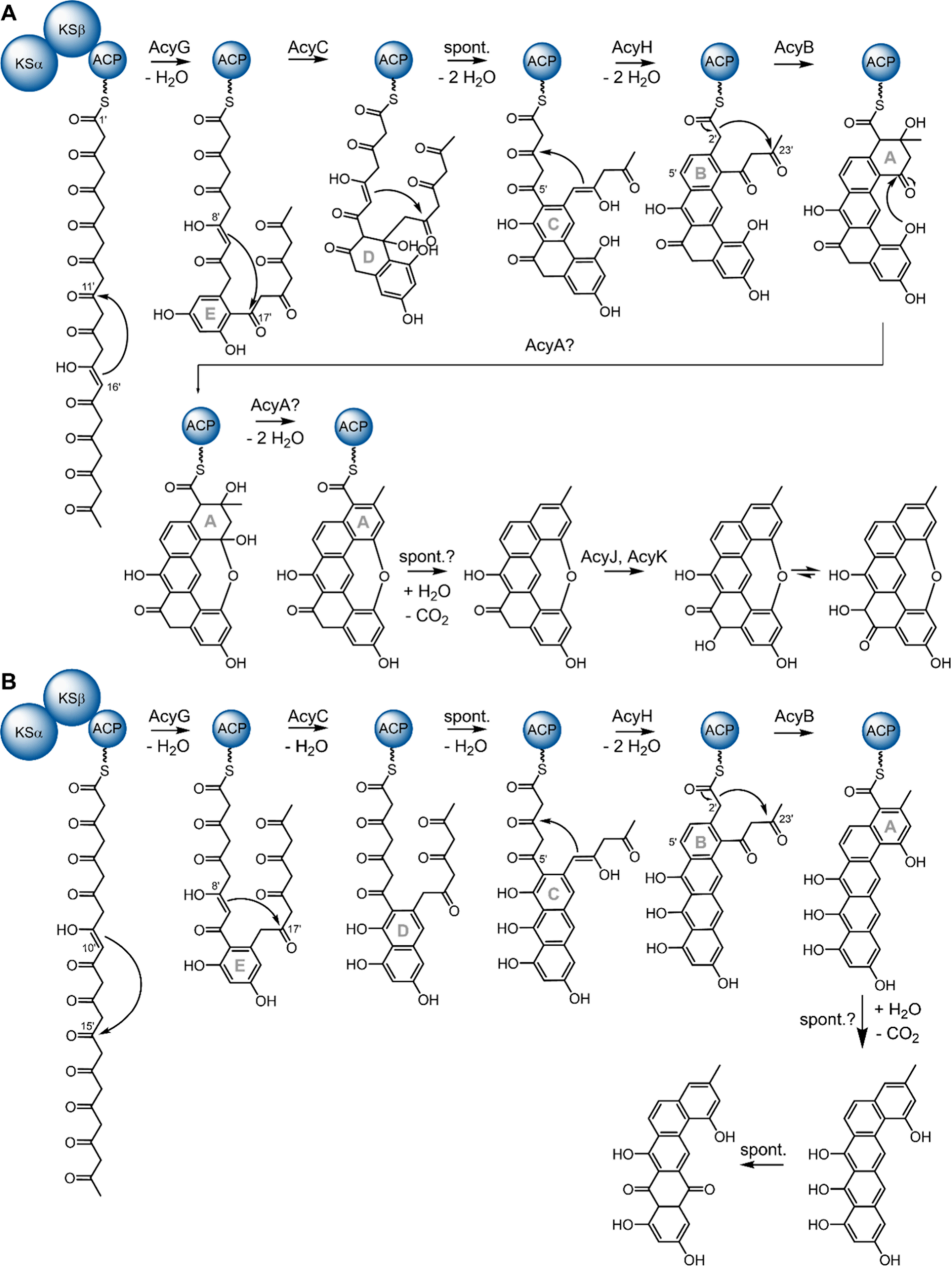
Aggregicyclin and oxyaggregicyclin biosynthesis. (A) Proposed biosynthesis route for aggregicyclin formation. (B) Proposed biosynthesis route for oxyaggregicyclin formation (KS, ketosynthase; ACP, acyl carrier protein; KR, ketoreductase; enzyme names with respect to Table S5).

The second ring cyclase AcyC shows homology to TcmJ and BenD (see Figure S20) and—based on the assigned function of TcmJ as second ring cyclase—likely is involved in formation of the D-ring during aggregicyclin biosynthesis by a C8′ to C17′ cyclization.^33^ Notably, the combined action of first and second ring cyclases also leads to the angucycline-type connection of the bent E, D, and C ring connection of the carbon skeleton as it is seen in landomycin.^36^

Third ring closure to form the C ring is often spontaneous in these biosyntheses as, e.g., observed in pyxidicyline formation.^11^ As we do not find a third ring cyclase in the acy BGC, we assume this to also be the case during aggregicyclin biosynthesis (Figure 4A and Tables S4 and S5).

The subsequent B-ring formation in aggregicyclin is intriguing from two perspectives: First, the biosynthesis introduces a second bent angucycline-like C–B–A ring junction, which is thermodynamically less favorable than a linear C–B–A ring system. Second, the final product lacks the phenolic hydroxy group on the D ring that would be expected upon direct aromatization, indicating that carbonyl reduction must occur during this step. The most plausible explanation for these observations is that the ketoreductase AcyH reduces the growing polyketide chain at the C5′ position to an alcohol, thereby preorganizing the intermediate for a bent angucycline-type aldol condensation and subsequent aromatization leading to D-ring closure. This mechanism would account not only for the observed ring connectivity but also for the absence of the phenolic hydroxy group on the D ring in the final product, which would be eliminated during the aromatization process.

The last cyclization reaction is likely performed by AcyB, which shows homology to late-stage cyclases such as TcmI and BenE (Figure 4A, Figure S20, and Tables S4 and S5).^33^ In the released aggregicyclin, the A and E rings are connected by a wide-spanning biaryl ether bridge, which is likely to be difficult to form once the A ring has aromatized. Therefore, it is plausible that the corresponding hemiacetal intermediate forms prior to the A-ring aromatization. Enzymes containing oxyanion holes—such as the hydrolase-type enzyme encoded by *acyA*—may be capable of catalyzing this reaction; however, follow-up in vitro experiments will be required to elucidate the mechanism underlying formation of these wide-spanning biaryl ether linkages although substrate availability limits such options.

After aromatization of the A ring, the compound is released by hydrolysis of the thioester bond to the carrier protein and subsequently decarboxylates (Figure 4A). This reaction might either occur spontaneously or could be part of an oxidative decarboxylation process that is not yet elucidated. The polyketide monooxygenase enzymes AcyJ and AcyK are finally responsible for installing the last oxygen atom onto the scaffold to complete the aggregicyclin molecule (Figure 4A and Tables S4 and S5).

Although closely related to aggregicyclin biosynthesis, the formation of oxyaggregicyclin differs in the stage of the first-ring closure (Figure 4B). Instead of a C11′–C16′ linkage, aromatization occurs between C10′ and C15′ (Figure 3C). We propose that oxyaggregicyclin is a byproduct of aggregicyclin biosynthesis, arising from mispairing of carbon atoms during the initial aldol condensation and aromatization step, due to either cyclase AcyG promiscuity or a spontaneous side reaction not controlled by the first-ring cyclase.

The following C8′–C17′ cyclization performed by AcyC results in a nonbent E–D–C ring system (Figure 4B). Subsequent closure and aromatization of ring C proceed analogously to aggregicyclin biosynthesis, including proposed reduction-triggered D-ring closure and aromatization. As in aggregicyclin formation, reduction at C5′ by the ketoreductase AcyH is required, consistent with the absence of a phenolic hydroxy group in the final oxyaggregicyclin product, which is removed via reduction followed by dehydration during aromatization (Figure 4B). Often, the ketoreduction in type II biosynthesis is hypothesized to be preorienting for the cyclization reactions, so the reaction of AcyH at C5′ may be the first reaction step followed by the cyclizations to form the oxyaggregicyclin backbone.

Finally, A-ring cyclization and aromatization are catalyzed by AcyB, a C2′–C23′ cyclase/aromatase, in a manner analogous to aggregicyclin biosynthesis (Figure 4B). Again, analogous to aggregicyclin biosynthesis, we observe hydrolytic release of the precursor molecule and either spontaneous or oxidative decarboxylation of the oxyaggregicyclin precursor. As a final step, the D ring of oxyaggregicylin is oxidized to form the corresponding anthraquinone structure. It is likely that this reaction is spontaneous, as it is described for anthraquinone-type molecules like the pyxidicyclines. These anthracycline-type C, D, and E ring connections of three phenolic rings are usually prone to oxidation.^11^ Taken together, we were able to assign plausible functions to all proteins encoded by genes of the *acy*BGC, providing a coherent biosynthesis hypothesis.

### 3.5 Aggregicyclins Show Broad Spectrum Inhibitory Activities

After evaluation of the biosynthesis of the aggregicyclins, we were interested in their biological activities with a special emphasis on putative antimicrobial or cytotoxic properties. While oxyaggregicyclin did not exhibit any significant antimicrobial activity in our assays (Tables S10 and S11), we could show antimicrobial activity for aggregicyclin. Aggregicyclin displays a minimal inhibitory concentratiosn (MIC) value of 22 μM against *Staphylococcus aureus* Newman and 86 μM against *Bacillus subtilis*, indicating them to have weak biological activity against Gram-positive bacteria.

More interestingly, aggregicyclin showed half maximal inhibitory (IC_50_) values against human cancer cells at low micromolar concentration at 1.2 and 1.8 μM against HepG2 and HCT116 cell lines, respectively, and slightly weaker cytotoxicity against KB3.1 cells at 11 μM. Unfortunately, the yield of oxyaggregicyclin was significantly lower compared to aggregicyclin, and it was therefore not tested against human cancer cells in our biological activity evaluation.

## 4 Conclusions

In this study, we demonstrate that *Myxococcota*—despite harboring comparatively few type II PKS systems—represents a previously underexplored source of chemically and biosynthetically distinctive type II polyketides. Through a systematic genome-mining survey, we identified a rare and compact type II PKS BGC from *A. edonensis* MCy10622 and successfully activated this otherwise cryptic pathway by heterologous expression in *M. xanthus*. This strategy enabled the discovery of the aggregicyclins, previously unknown type II polyketides, and underscored the power of targeted cluster refactoring and heterologous expression approaches for unlocking the biosynthetic potential in noncanonical producers. Notably, we were not able to detect aggregicyclin production in the wild-type strains harboring an *acy*BGC.

Structural elucidation revealed that aggregicyclin and oxyaggregicyclin possess markedly divergent cyclization patterns despite originating from the same biosynthetic machinery. Our biosynthetic analysis supports an early diversification event during first-ring formation as the key determinant of this divergence, highlighting how subtle variations in cyclase specificity or timing can generate pronounced structural diversity in iterative PKS systems. The aggregicyclins further expand the known chemical space of type II polyketides by featuring an unusual, wide-spanning biaryl ether linkage, a motif that has not been observed in natural products and presents a challenging motif for incorporation through synthetic chemistry.

Beyond their structural novelty, aggregicyclins exhibit biologically relevant activities. Aggregicyclin shows weak antibacterial activity against Gram-positive bacteria and moderate cytotoxicity against human cancer cell lines, positioning it as an unprecedented scaffold for further pharmacological exploration, albeit with challenges related to chemical instability. These findings reinforce the notion that chemically fragile or transient metabolites—often overlooked due to isolation difficulties—may nonetheless play important ecological or biological roles. Based on the observed inhibitory activities against bacteria and human cells, the aggregicyclins may serve as defensive molecules for their producing organism. However, their precise role in an ecological context remains unclear and, therefore, an interesting starting point for future studies.

Taken together, our work expands the currently sparse knowledge of type II polyketides from Gram-negative bacteria and their biosynthetic machineries and establishes *Myxococcota* as a valuable source of unconventional type II PKS chemistry. More broadly, it illustrates how integrating genome mining, synthetic biology–based pathway activation, and detailed biosynthetic analysis can uncover novel natural products and biosynthetic logic from underexplored bacterial phyla, thereby opening new avenues for natural product discovery.

## Supplementary Material



## Notes

Bacterial growth and fermentation conditions; metabolite extraction procedures; UHPLC–MS methods and metabolomics data processing; in silico analyses of the *acy*BGC; cloning and heterologous expression protocols; primer sequences; isolation and purification procedures; NMR-based structure elucidation and spectra; biological assay conditions (PDF)
