# Supplementary material for: Aggregicyclins Shed Light on Type II Polyketide Biosynthesis in Myxococcota
Source: JACS Au. 2026 Apr 29;6(5):2924–34. doi: 10.1021/jacsau.6c00256 (PMC13213394; doi:10.1021/jacsau.6c00256)
Supplement: Supplementary file 1 [file au6c00256_si_001.pdf]

Supporting Information

Aggregicyclins Shed Light on Type II Polyketide Biosynthesis in *Myxococcota*

Chantal D. Bader [a,c]†, Sophia Panter [a]†, Fabian Panter [a]†, Amay Ajaykumar Agrawal[b,e], Olga V. Kalinina[b,c,e,f], and Rolf Müller* [a,c,d]

[a] Department of Microbial Natural Products, Helmholtz-Institute for Pharmaceutical Research Saarland (HIPS), Helmholtz Centre for Infection Research (HZI) and Department of Pharmacy, Saarland University, Campus E8 1, 66123 Saarbrücken, Germany

[b] Research Group Drug Bioinformatics, Helmholtz Institute for Pharmaceutical Research Saarland (HIPS), Helmholtz Centre for Infection Research (HZI), 66123, Saarbrücken, Germany

[c] PharmaScienceHub, Saarbrücken, Germany

[d] German Centre for Infection Research (DZIF), Partner Site Hannover–Braunschweig, Germany

[e] Center for Bioinformatics, Saarland University, Saarbrücken, Germany

[f] Medical Faculty, Saarland University, Homburg, Germany

† These authors contributed equally.

Table of Contents

1 BACTERIAL GROWTH CONDITIONS 3

1.1 BACTERIAL CULTURE MEDIA 3
1.2 MYXOBACTERIAL FERMENTATION CONDITIONS FOR LC-MS ANALYSIS 4
1.3 LARGE SCALE PRODUCTION OF *M. XANTHUS* DK1622::PSKT2PKS FOR AGGREGICYCLIN ISOLATION 4

2 ANALYTICAL METHODS USED IN THIS WORK 5

2.1 METABOLITE EXTRACTION PROCEDURE FOR ANALYTICAL SCALE EXTRACTIONS 5
2.2 STANDARDIZED UHPLC MS CONDITIONS 5
2.3 METHODOLOGY FOR STATISTICS-BASED METABOLOME FILTERING 6
2.4 ACQUISITION PARAMETERS FOR ACQUIRING OF TARGETED HIGH-RESOLUTION TANDEM MS DATA . 6

3 *IN-SILICO* ANALYSIS OF THE AGGREGICYCLIN (*ACY*) BIOSYNTHETIC GENE CLUSTER (BGC) 7

3.1 PREPARATION OF GENOMIC DNA FOR PACBIO SEQUENCING OF MCY10622 7
3.2 OVERVIEW OVER THE *ACY* BIOSYNTHETIC GENE CLUSTER FROM MCY10622 8
3.3 *IN-SILICO* BLAST ANALYSIS OF THE *A. EDONENSIS* MCY10622 AGGREGICYCLIN BIOSYNTHETIC GENE CLUSTER 9
3.4 *IN-SILICO* BLAST ANALYSIS OF THE PUTATIVE *M. BOLETUS* MEB2 AGGREGICYCLIN BIOSYNTHETIC GENE CLUSTER 10

4 CLONING AND HETEROLOGOUS EXPRESSION OF THE *ACY* BGC 11

4.1 LIST OF PRIMERS USED IN THIS STUDY 11
4.2 PCR REACTIONS AND CYCLER PROTOCOLS 12
4.2.1 Thermo scientific Phusion Polymerase 12
4.3 PROTOCOLS FOR Λ-RED PROPHAGE RECOMBINATION (RED/ET) 13
4.4 CREATION OF THE PSKT2PKS CLUSTER PLASMID COVERING THE ACY BGC FOR SINGLE CROSSOVER INTEGRATION 14
4.5 TRANSFORMATION OF *MYXOCOCCUS XANTHUS DK1622* 18
4.5.1 Transformation protocol for M. xanthus DK1622 18

5 ISOLATION AND STRUCTURE ELUCIDATION OF THE AGGREGICYCLINS 19

5.1 PURIFICATION OF AGGREGICYCLINS BY HPLC 19
5.2 NMR BASED STRUCTURE ELUCIDATION 22

6 BIOSYNTHESIS OF THE AGGREGICYCLINS 31

7 NMR SPECTRA EMPLOYED IN AGGREGICYCLIN STRUCTURE ELUCIDATION 32

8 BIOLOGICAL ASSAY CONDITIONS 46

9 REFERENCES 47

1 Bacterial growth conditions

1.1 Bacterial culture media

Table S 1. Recipe for CTT medium for Myxococcus xanthus DK1622

CTT – Medium			
Amount	Ingredient	Concentration	Supplier
10 g/L	Casitone	-	BD
1 mL/L	$KHPO_4$ • pH 7.6	0.1 M	Sigma Aldrich
10 mL/L	$MgSO_4$ • $7H_2O$	0.8 M	Grüssing
10 mL/L	TRIS • HCl pH8	1 M	Sigma Aldrich
100 µL/L	Sterile Vit. B12 solution (added after autoclaving)	1 mg/mL	Roth
200 µL/L	Sterile FeEDTA solution (added after autoclaving)	8 mg/mL	Sigma Aldrich
Dissolved in milli-Q. Water, pH adjusted to 7.6 with 1 N KOH			

Table S 2. Recipe for LB medium for E. coli DH10β

LB – Medium			
Amount	Ingredient	Concentration	Supplier
5 g/L	Yeast extract	-	Roth
10 g/L	Tryptone	-	Roth
10 g/L	Starch (soluble)	-	Roth
5 g/L	NaCl	-	Sigma Aldrich
Dissolved in milli-Q. Water, pH adjusted to 7.2 with 1 N KOH			

Table S 3: Medium recipe of VYGS medium for Aggregicoccus edonensis MCy10622(Sood *et al.*, 2015)

VYGS – Medium			
Amount	Ingredient	Concentration	Supplier
10 g/L	Baker's Yeast	-	Lynco
5 g/L	Glucose	-	Roth
10 g/L	Starch (soluble)	-	Roth
1 g/L	$CaCl_2$	-	Sigma Aldrich
1 g/L	$MgSO_4$ • $7H_2O$	-	Grüssing
10 mM	TRIS • HCl pH8	-	Sigma Aldrich
100 µL/L	Sterile Vit. B12 solution (added after autoclaving)	1 mg/mL	Roth
200 µL/L	Sterile FeEDTA solution (added after autoclaving)	8 mg/mL	Sigma Aldrich
50 µL/L	Sterile Riboflavin solution (added after autoclaving)	1 mg/mL	Roth
Dissolved in milli-Q. water pH adjusted to 7.2 with 1 N KOH			

The myxobacterial strain *M. xanthus* DK1622 was kept in agar culture both for storage over short amounts of time and for cloning. The agar media used for *M. xanthus* are CTT agar and CTT soft agar, which is prepared by adding 14 g/L agarose and 8 g/L agarose (BD) to CTT medium preparations before autoclaving. The myxobacterial strain *A. edonensis* MCy10622 was kept in agar culture on VYGS agar for storage over short amounts of time. For propagation for genomic DNA extraction, it was cultivated in VYGS liquid medium. All manipulations of plasmids are done in *E. coli* DH10β cultivated in Lysogeny Broth (LB) medium (Table S2) or on Luria Bertone Agar prepared by adding 14 g/L agarose to LB medium before autoclaving.

1.2 Myxobacterial fermentation conditions for LC-MS analysis

Cultures for UHPLC-*hr*MS analysis are grown in 300 mL shake flasks containing 50 mL of CTT medium for *M. xanthus* DK1622 inoculated with 1 mL of pre culture. *A. edonensis* cultures are grown in 300 mL shake flasks containing 50 mL of VYGS medium inoculated with 1 mL of pre culture. Media for mutant DK1622 strains were supplemented with 12 mg/L oxytetracycline hydrochloride (Sigma Aldrich) in 70% EtOH and 1 mM of aqueous sterile filtrated potassium vanillate solution if the corresponding strain's vanillate promotor is to be induced. Since aggregicyclin seems to bind to the van repressor, leading to auto induction, which also means that production of aggregicyclin is not affected by potassium vanillate induction, we did not induce most of the aggregicyclin fermentation cultures. After inoculation the medium is supplemented with 2% of sterile XAD-16 adsorber resin (Sigma Aldrich) suspension in water to bind secondary metabolites in the culture medium and limit aggregicyclin autotoxicity. Small scale cultures were grown for 10-12 days wrapped in aluminum foil to keep out residual light. After fermentation the culture XAD-16 mixture is pelleted in a 50 mL falcon at 6000 rcf for 10 minutes using an Eppendorf falcon table centrifuge and stored at -20 °C until further use. Cell pellet and cultures are shielded from light as much as possible due to the light sensitivity of the aggregicyclins.

1.3 Large scale production of *M. xanthus* DK1622::pSKt2PKS for Aggregicyclin isolation

The *M. xanthus* strain DK1622::pSKt2PKS is fermented in 50 mL CTT medium as a seed culture flasks on an Orbiton shaker at 160 rpm and 30 °C. The translucid culture medium becomes brownish/yellow and opaque after 4 to 7 days of fermentation. This pre-culture is used to inoculate 6 x 2 L CTT medium supplemented with 2% XAD-16 resin suspension in sterilized water in 6 x 5 L baffled shake flasks on an Orbiton shaker at 160 rpm and 30 °C. The shake flasks are wrapped in aluminum foil to keep out the majority of light radiation from the culture to keep the aggregicyclins from degrading. Fermentation is complete after 8 days. Cells and XAD-16 resin are harvested by centrifugation on a Beckmann Avanti J-26 XP with the JLA 8.1 rotor at 6000 rcf. Combined resin and cells are freeze dried and subsequently extracted using 2x 500 mL of methanol (technical grade, Fluka) and subsequently 500ml of acetone (technical grade, Fluka). The combined extracts are concentrated on a rotary evaporator and partitioned between methanol and hexane. The aggregicyclins remain in the methanol phase. The methanol phase is dried with a rotary evaporator and the residue is partitioned between water and chloroform. As the aggregicyclins are neither soluble in Water nor in chloroform they precipitate as a blackish yellowish amorphous solid. After filtering of the solvent through glass wool that retains the aggregicycline containing precipitate, the aggregicyclins are taken up in methanol, centrifuged and stored at -20°C for HPLC separation in an aluminum foil wrapped glass vial. During the whole extraction process both the cell pellet as well as all the fraction from liquid-liquid extraction are carefully protected from light as well as possible.

2 Analytical methods used in this work

2.1 Metabolite extraction procedure for analytical scale extractions

The frozen cell pellet from an analytical scale culture is transferred into a 100 mL Erlenmeyer flask and a magnetic stirrer is added. 50 mL of acetone (Fluka analytical grade, redistilled in house) are added onto the pellet and the mixture is stirred for 60 min on a magnetic stirrer. The acetone extract is left to settle in order to sediment cell debris and XAD resin for a second extraction step. The supernatant is filtered with a 125-micron folded filter keeping cell pellet and XAD-16 resin in the Erlenmeyer flask for a second extraction step. The residual pellet and XAD-16 resin is extracted again with 30 mL of distilled acetone for 60 min on a magnetic stirrer and filtered through the same folded filter. The combined extracts are transferred into a 100 mL round bottom flask. The acetone is evaporated using a rotary evaporator at 260 mbar and 40 °C water bath temperature. The residual water is evaporated at 20 mbar until the residue in the flask is completely dry. The residue is taken up in 550 µL of methanol (Chromasolv HPLC grade, Sigma Aldrich) and transferred into a 1.5 mL Eppendorf tube. This tube is centrifuged with a Hitachi table centrifuge at 15000 rpm for 2 minutes to remove residual insolubilities such as salts, cell debris and XAD fragments. The residual extract is diluted 1:10 for UHPLC-*hr*MS analysis.

2.2 Standardized UHPLC MS conditions

UPLC-*hr*MS analysis is performed on a Dionex (Germering, Germany) Ultimate 3000 RSLC system using a Waters (Eschborn, Germany) BEH C18 column (50 x 2.1 mm, 1.7 µm) equipped with a Waters VanGuard BEH C18 1.7 µm guard column. Separation of 1 µL sample is achieved by a linear gradient from (A) H_2O + 0.1 % FA to (B) ACN + 0.1 % FA at a flow rate of 600 µL/min and a column temperature of 45 °C. Gradient conditions are as follows: 0 – 0.5 min, 5% B; 0.5 – 18.5 min, 5 – 95% B; 18.5 – 20.5 min, 95% B; 20.5 – 21 min, 95 – 5% B; 21-22.5 min, 5% B. UV spectra are recorded by a DAD in the range from 200 to 600 nm. The LC flow is split to 75 µL/min before entering the Bruker Daltonics maXis 4G *hr*ToF mass spectrometer (Bremen, Germany) equipped with an Apollo II ESI source. Mass spectra are acquired in centroid mode ranging from 150 – 2500 m/z at a 2 Hz full scan rate. Mass spectrometry source parameters are set to 500 V as end plate offset; 4000 V as capillary voltage; nebulizer gas pressure 1 bar; dry gas flow of 5 l/min and a dry temperature of 200 °C. Ion transfer and quadrupole settings are set to funnel RF 350 Vpp.; multipole RF 400 Vpp as transfer settings and ion energy of 5 eV as well as a low mass cut of 300 m/z. Collision cell is set to 5.0 eV and pre-pulse storage time is set to 5 µs. Spectra acquisition rate is set to 2 Hz. Calibration is done automatically before every LC-MS run by injection of sodium formate and calibration on the respective clusters formed in the ESI source. All MS analyses are acquired in the presence

of the lock masses ($C_{12}H_{19}F_{12}N_3O_6P_3$, $C_{18}H_{19}F_{24}O_6N_3P_3$ and $C_{24}H_{19}F_{36}N_3O_6P_3$), which generate the $[M+H]^+$ ions of 622.0289; 922.0098 and 1221.9906.

2.3 Methodology for statistics-based metabolome filtering

In order to detect all metabolites appearing after heterologous expression of the *acy* BGC we compare wild-type *M. xanthus* DK1622 to the aggregicyclin cluster heterologous expression mutant *M. xanthus* DK1622::pSKt2PKS in an unbiased principal component analysis (PCA) based statistical analysis adapted from Panter et al..(Panter *et al.*, 2019) For this purpose, LC-MS chromatograms of 3 independent cultivations are measured as 2 technical replicates each, giving a total number of 6 LC-*hr*MS chromatograms per strain. To obtain all molecular features in the 6 LC-*hr*MS chromatograms of the bacterial extracts of the induced mutant strains and the 6 LC-*hr*MS chromatograms of the corresponding wild type strain extracts, the T-ReX-3D molecular feature finder implemented in Bruker Metaboscape 5 is used. Compound detection parameters intensity threshold is set to 10000, m/z threshold to 0.005 Da and minimum compound length to 4 spectra. PCA t-test tables are created with the built in PCA t-test routine and filtered according to 6 appearances in the *acy* cluster heterologous expression mutant extract chromatograms and 0 appearances in the DK1622 wild type extract chromatograms. The t-test table from the bucketing will be provided upon request.

2.4 Acquisition parameters for acquiring of targeted high-resolution tandem MS data

LC and MS conditions for SPL guided MS/MS data acquisitions are kept constant according to section standardized UHPLC-MS conditions. MS/MS data acquisition parameters are set to exclusively fragment scheduled precursor list entries. SPL tolerance parameters for precursor ion selection are set to 0.2 minutes and 0.05 m/z in the SPL MS/MS method. The method picks up to 2 precursors per cycle, applies smart exclusion after 5 spectra and performs CID and MS/MS spectra acquisition time ramping. CID Energy is ramped from 35 eV for 500 m/z to 45 eV for 1000 m/z and 60 eV for 2000 m/z. MS full scan acquisition rate is set to 2 Hz and MS/MS spectra acquisition rates are ramped from 1 to 4 Hz for precursor ion intensities of 10 kcts. to 1000 kcts..

3 *In-silico* analysis of the aggregicyclin (*acy*) biosynthetic gene cluster (BGC)

3.1 Preparation of genomic DNA for PacBio sequencing of MCy10622

To isolate total DNA for sequencing purposes such as PacBio sequencing, phenol-chloroform gDNA extraction is used.

1) Spin down 50 mL of fresh myxobacterial culture 6000 rcf 10 min
2) Discard the supernatant
3) Wash the cells once with SET Buffer, centrifuge at 6000 rcf 10 min
4) Resuspend cell pellet in 5 mL SET Buffer
5) Add 100 µL of lysozyme (50 mg/mL in ddH_2O) stock solution as well as 50 µL RNAse A (10 mg/mL in ddH_2O) stock solution
6) Add 300 µL Proteinase K solution (10 mg/mL 50 mM Tris 1 mM $CaCl_2$) invert several times and add 600µL 10% SDS solution
7) Incubate at 55 °C for 2 h, invert every 15 min
8) Add even Volume (6 mL) of Phenol/Chloroform/Isoamylalcohol (25:24:1) and swing the tube for 60 min at 5 rpm
9) Centrifuge the mixture at 6000 rcf for 5 min at room temperature
10) Transfer the upper phase into a new tube using a cut end 1 mL tip
11) Add even Volume (6 mL) of Phenol/Chloroform/Isoamylalcohol (25:24:1) and swing the tube for 60 min at 5 rpm
12) Centrifuge the mixture at 6000 rcf for 5 min at room temperature
13) Transfer the upper phase into a new tube using a cut end 1 mL tip
14) Add even Volume (6 mL) of Chloroform/Isoamylalcohol (24:1) and swing the tube for 60 min at 5 rpm
15) Centrifuge the mixture at 6000 rcf for 5 min at room temperature
16) Transfer the upper phase into a new tube using a cut end 1 mL tip
17) Add 1/10 of the total volume of 3 M NaOAc solution pH 5.5 and mix well by inverting several times
18) Add 2.5 Volumes of ice-cold ethanol (100% technical purity, -20 °C) and invert the tube several times, DNA precipitation should be visible as a cotton like fog in the tube
19) Spool the DNA on a sealed Pasteur pipette
20) Rinse the DNA with 70% Ethanol (cold, -20 °C)
21) Air dry the DNA for at least 15 minutes (Dry DNA will become completely translucid)
22) Resuspend dried DNA in 0.5 mL of ddH_2O and keep the Eppendorf tube at room temperature for 24 Hours

3.2 Overview over the *acy* biosynthetic gene cluster from MCy10622

Overview about the cloned aggregicyclin BGC as found in *A. edonensis* MCy10622 is depicted in Figure S 1.

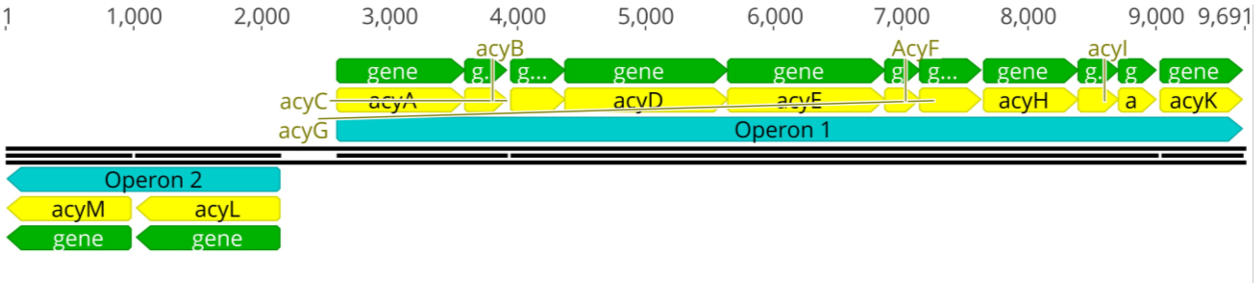

Figure S 1. Schematic view of the MCy10622 acy biosynthetic gene cluster including gene names and gene lengths

Overview about the aggregicyclin BGC as found in *Melittangium boletus* Meb2 is depicted in Figure S 2.

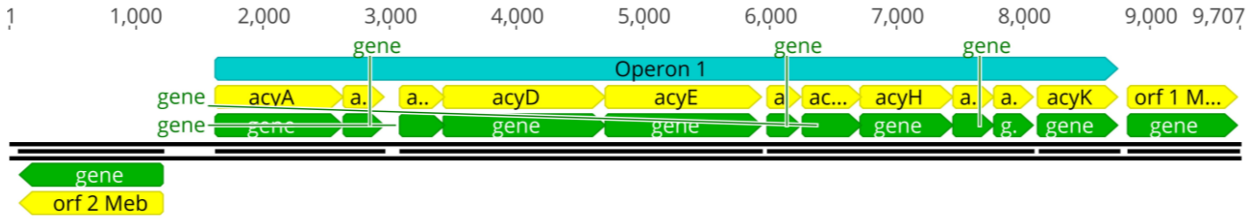

Figure S 2. Schematic view of the Meb2 acy biosynthetic gene cluster including gene names and gene lengths

The genome sequence of *Aggregicoccus edonensis* MCy10622 will be made available in NCBI and the *acy*BGC in MiBiG upon publication of the manuscript.

3.3 *In-silico* blast analysis of the *A. edonensis* MCy10622 aggregicyclin biosynthetic gene cluster

Every coding sequence in the Mcy10622 aggregicyclin biosynthesis gene cluster (*acy* cluster) was extracted translated and searched with the blastp algorithm against the RefSeq non-redundant protein sequence database at NCBI. (O'Leary *et al.*, 2016)

Table S 4. Tabulated blastP results for the CDS regions present in the MCy10622 acy biosynthetic gene cluster

CDS Name	Length [AA]	Closest homologue [Organism of origin]	Identity [%] and alignment length [AA]	Proposed function	Accession Nr.
acyA	333	amidohydrolase family protein [Streptomyces thermoautotrophicus]	60.6, 324	-	WP_067068858
acyB	108	TcmI family type II polyketide cyclase [Melittangium boletus]	69.4, 98	Polyketide cyclization	WP_095982803
acyC	143	cupin domain-containing protein [Micromonospora sp. MW-13]	75.3, 110	-	WP_117669315
acyD	432	beta-ketoacyl ACP synthase family protein [Streptomyces thermoautotrophicus]	88.9, 422	KSα Ketosynthase	WP_067068863
acyE	411	ketosynthase chain-length factor [Melittangium boletus]	85.2, 397	KSβ chain length factor	WP_095982804
acyF	90	acyl carrier protein [Streptomyces zinciresistens]	55.0, 59	Acyl carrier protein	WP_007492795
acyG	159	SRPBCC family protein [Streptomyces thermoautotrophicus]	70.9, 148	Polyketide cyclization	WP_067068872
AcyH	244	3-oxoacyl-ACP reductase FabG [Melittangium boletus]	82, 244	Ketoreductase	WP_095978133
acyI	106	antibiotic biosynthesis monooxygenase [Melittangium boletus]	78.8, 104	Monooxygenase	WP_095978134
acyJ	99	antibiotic biosynthesis monooxygenase [Melittangium boletus]	64.4, 98	Monooxygenase	WP_095978135
acyK	210	SDR family oxidoreductase [Melittangium boletus]	71.1, 211		WP_095978136
acyL	376	alpha/beta hydrolase [Melittangium boletus]	69.1, 375	-	WP_095978127
acyM	324	ACP S-malonyltransferase [Archangium sp. Cb G35]	73.9, 307	-	WP_073563445

3.4 *In-silico* blast analysis of the putative *M. boletus* Meb2 aggregicyclin biosynthetic gene cluster

Every coding sequence in the Meb2 aggregicyclin biosynthesis gene cluster (*acy* cluster) was extracted translated and searched with the blastp algorithm against the RefSeq non-redundant protein sequence database at NCBI. (O'Leary *et al.*, 2016)

Table S 5. Tabulated blastP results for the CDS regions present in the Meb2 acy biosynthetic gene cluster

CDS Name	Length [AA]	Closest homologue [Organism of origin]	Identity [%] and alignment length [AA]	Proposed function	Accession Nr.
acyA Meb		amidohydrolase family protein [Streptomyces thermoautotrophicus]	60.6, 324	-	WP_067068858
acyB Meb		TcmI family type II polyketide cyclase [Melittangium boletus]	69.4, 98	Polyketide cyclization	WP_095982803
acyC Meb		cupin domain-containing protein [Micromonospora sp. MW-13]	75.3, 110	-	WP_117669315
acyD Meb		beta-ketoacyl ACP synthase family protein [Streptomyces thermoautotrophicus]	88.9, 422	KSα Ketosynthase	WP_067068863
acyE Meb		ketosynthase chain-length factor [Melittangium boletus]	85.2, 397	KSβ chain length factor	WP_095982804
acyF Meb		acyl carrier protein [Streptomyces zinciresistens]	55.0, 59	Acyl carrier protein	WP_007492795
acyG Meb		SRPBCC family protein [Streptomyces thermoautotrophicus]	70.9, 148	Polyketide cyclization	WP_067068872
AcyH Meb		3-oxoacyl-ACP reductase FabG [Melittangium boletus]	82, 244	Ketoreductase	WP_095978133
acyI Meb		antibiotic biosynthesis monooxygenase [Melittangium boletus]	78.8, 104	Monooxygenase	WP_095978134
acyJ Meb		antibiotic biosynthesis monooxygenase [Melittangium boletus]	64.4, 98	Monooxygenase	WP_095978135
acyK Meb		SDR family oxidoreductase [Melittangium boletus]	71.1, 211	-	WP_095978136
orf1 Meb		alpha/beta hydrolase [Melittangium boletus]	69.1, 375	-	WP_095978127
orf2 Meb		ACP S-malonyltransferase [Archangium sp. Cb G35]	73.9, 307	-	WP_073563445

4 Cloning and heterologous expression of the *acy* BGC

4.1 List of primers used in this study

Table S 6. List of all primers used for creation of the pSKt2PKS vector

Primers used for the creation of pSKt2PKS	
Primer name	Primer 5’ to 3’ sequence
VanR fwd HindIII	ATATAAGCTTGATTCAGTCGGCGCG
VanR rev NdeI	ATATGGAATTCGCATATGCGTTTCCTCG
Mx8 ApaLI fwd	ATATGTGCACGGCCATCGTCGAAAGAGTCG
Mx8 ApaLI rev	ATATGTGCACAGGACTCCTCTGGCTGGGTG
Operon 1 fwd NdeI	AAAACATATGATTATCGACATCCACTC
Operon 1 rev NdeI	ATATCCATATGGCTGAGAGGCTTCG
RedET Operon 2 fwd	GGTCATAGCTGTTTCCTGTGTGAAATTGTTATCCGCTCACAATTCCACAC AACTGAACAGGAGGGACAGCTG
RedET Operon 2 rev	TCCGCTTACAGACAAGCTGTGACCGTCTCCGGGAGCTGCATGTGTCAG AGGTCTACCTCGCTGCCTCCCG
T7A1 Operon 2 rev	GCCATCGAGAGGTGTACATGTGCTGAACCAGGATGTCC
T7A1 Operon 2 fwd	GGACATCCTGGTTCAGCACATGTACACCTCTCGATGGC
pSKt2PKS int fwd	GCCTTCCACATGACGGGGCT
pSKt2PKS int rev	GGTCCCCTTCATGCCGTGCT

4.2 PCR reactions and cycler protocols

4.2.1 Thermo scientific Phusion Polymerase

Phusion Polymerase PCR mix:

5 µL GC Buffer

2.5 µL dNTP's (2 mM)

1.25 µL DMSO

0.5 µL Primer fwd and rev (100mM)

0.2 µL Phusion DNA Polymerase

0.5 µL gDNA Template (~50 ng/µL)

14.55 µL ddH_2O

Phusion Polymerase cycler program:

Table S 7: Thermo Phusion DNA Polymerase PCR program

Step	Time [min:s]	Temperature [°C]
**Initial denaturation**	4:00	95
	0:30	98
**Cycle, repeat 30x**	0:15	63
	0:30 per Kbp to amplify	72
**Final elongation**	10	72
**Store**	forever	8

4.3 Protocols for λ-Red prophage recombination (Red/ET)

The strain used for all λ-Red based recombination experiments was *E. coli* GB08-red carrying the red γβαA genes in its bacterial chromosome.

Incorporation of the target plasmid into the λ-Red recombination strain

1) Inoculate 25 ml LB containing and grow the strain overnight at 30°C 200 rpm on a rotary shaker
2) Inoculate 20 µL of the well grown *E. coli* overnight culture into a 2 ml Eppendorf tube with a punctured lid containing 1.5 ml LB
3) Incubate the culture for 4h until OD reaches approx. 0.6
4) Mix target vector or Ligation mixture (ca.1-3µL) with competent *E. coli* and pipet the mixture into a Electroporation cuvette (100µL)
5) Electroporate the mixture at 1350V, 10µF, 600 Ohm, 1mm Cuvette length
6) Add 1mL fresh sterile LB medium, incubate for 1h at 900 rpm 30°C in a 2ml Eppendorf tube with a punctured lid
7) Plate on LB Oxytet12 Agar (provided the plasmid contains Oxytetracycline resistance, if not use appropriate antibiotic) and incubate the plates overnight at 30°C
8) Pick around 6 clones and cultivate them for plasmid preparation via alkaline lysis
9) Confirm the electroporated plasmid via restriction digestion

Modification of plasmids via Red/ET

1) Inoculate 25 ml LB containing 12 µg/ml oxytetracycline and grow the strain overnight at 30°C 200 rpm on a rotary shaker
2) Inoculate 20 µL of the well grown *E. coli* overnight culture into a 2 ml Eppendorf tube with a punctured lid containing 1.5 ml LB with 12 µg/ml oxytetracycline
3) Incubate the culture for 2h until OD reaches approx. 0.2 (sometimes longer incubation times are necessary)
4) Centrifuge the culture at 8000 x g for 5 minutes to pellet the cells
5) Wash the residual cell pellet 2 times with 1000 µL autoclaved milliqH_2O and discard the supernatant.
6) Re suspend the cell pellet in 50 µL autoclaved milliq H_2O and add the PCR product for Red/ET based modification
7) Electroporate the mixture at 1300V, 25µF, 600 Ohm, 1mm Cuvette
8) Add 1mL fresh sterile LB medium, incubate for 1h at 900 rpm 30°C in a 2ml Eppendorf tube with a punctured lid
9) Transfer the mixture into 50 ml LB containing appropriate concentration of the antibiotic on the modification PCR product (in this case Chloramphenicol 25µg/ml) and incubate over night at 30°C
10) Prepare plasmid from this mixture via alkaline lysis

11) Electroporate the plasmid mix into *E. coli DH10ß* according to the *E. coli DH10ß* electroporation protocol and plate it on LB Oxytet12 Cm25
12) Pick around 6 clones and cultivate them for plasmid preparation via alkaline lysis
13) Confirm the electroporated plasmid via restriction digestion

The retransformation is necessary in order to avoid having clones harboring both plasmids (the modified and the unmodified one) in the same *E. coli* clone and transfer the modified plasmid into the *E. coli* cloning strain DH10ß.

4.4 Creation of the pSKt2PKS cluster plasmid covering the acy BGC for single crossover integration

The plasmid is based on the pFPtetp15A *pcyJ* plasmid used for overexpression of the second operon of the pyxidicycline biosynthesis cluster in *Pyxidicoccus fallax* An d48.(Panter *et al.*, 2018) The plasmid solely contains a Ptet promoter controlled tetracycline efflux pump (*tetR*), a p15A replication origin and a Ptn5 promotor controlled *pcyJ* gene from the pyxidicycline pathway.

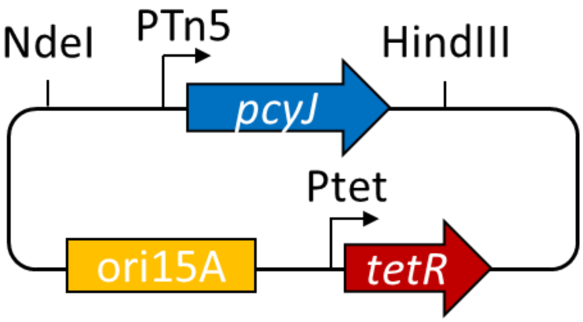

Figure S 3. Schematic depiction of pFPtetp15A pcyJ

First, in order to be able to transfer the plasmid at hand into *M. xanthus* DK1622 we replaced the *pcyJ* gene by a vanillate promotor and repressor system (*vanR*/PVan) to be able to induce aggregicyclin production in the heterologous host.(Iniesta *et al.*, 2012) The resulting plasmid was called pFPVanp15A. Correct cloning was confirmed by restriction digestion followed by agarose gel electrophoresis.

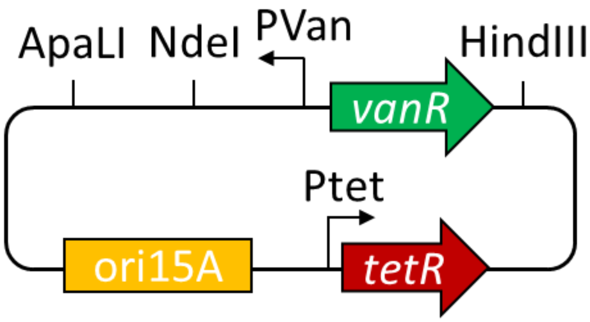

Figure S 4. Schematic depiction of the pFPVanp15A plasmid

As a next step, we added the Mx8 integrase gene from the myxobacterial Mx8 prophage that can mediate transfer of the plasmid into the Mx8 attB site of *M. xanthus* DK1622 to create the plasmid pFPVanp15A Mx8.(Orndorff *et al.*, 1983; Magrini *et al.*, 1999) Therefore, the Mx8 integrase is PCR amplified using the described PCR Phusion polymerase protocol and the primers Mx8 ApaLI fwd and Mx8 ApaLI rev from the pCIY Pyxidicycline cluster plasmid also used in the heterologous expression of the Pyxidicyline biosynthetic gene cluster.(Magrini *et al.*, 1999; Panter *et al.*, 2018) Cloning is performed by standard restriction ligation using the restriction endonuclease ApaLI. Selection of clones featuring the correct orientation of the Mx8 gene was done by restriction digestion followed by agarose gel electrophoresis.

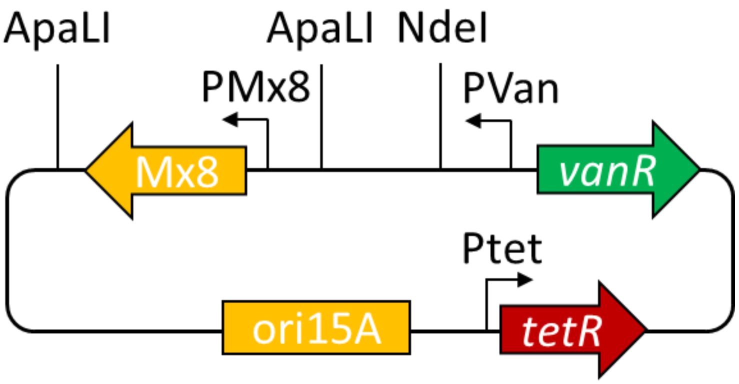

Figure S 5. Schematic depiction of the pFPVanp15A Mx8 plasmid

Following this restriction cloning step, we attempted to amplify the entire operon 1 spanning the genes *acyA* to *acyK* on 7110 bp. This was done by PCR using the Phusion PCR protocol and the entire operon was PCR amplified in one step using the primers Operon 1 fwd NdeI and Operon 1 rev NdeI. The operon is entered into the plasmid by restriction ligation using the restriction endonuclease NdeI and selection of clones having correct orientation of operon 1 by restriction analysis followed by agarose gel electrophoresis. Correct clones that were showing the integration of operon 1 in the right orientation are clones 36, 67 and 68.

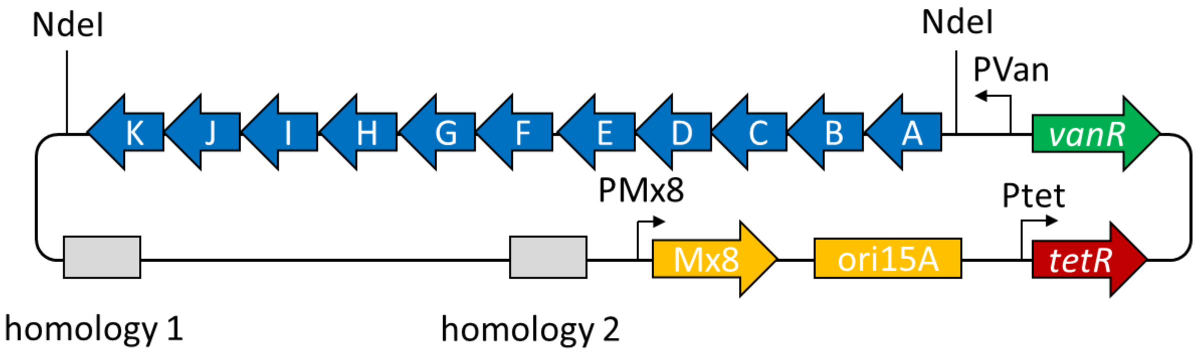

Figure S 6. Schematic depiction of the pSK Operon1 plasmid

To integrate operon 2, we chose λ-red prophage mediated recombination engineering (Red/ET) in the *E. coli* strain *E. coli* GB08-red. Therefore, a Chloramphenicol acetyl transferase (*cat/cmR*) gene fused to a T7A1 promoter is PCR amplified using the Primers RedET Operon 2 fwd and T7A1 Operon2 rev as well as the aggregicyclin operon 2 consisting of the genes *acyL* and *acyM* using the primers RedET Operon 2 rev and T7A1 Operon2 fwd. The two PCR products are fused by overlap extension PCR using the homology arm containing Primers RedET Operon 2 fwd and RedET Operon2 rev.

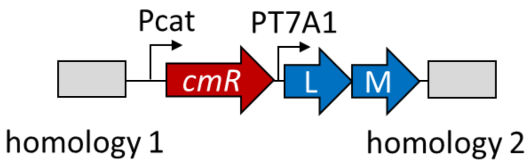

Figure S 7. Schematic depiction of Overlap extension PCR product for integrating Operon 2 via Red/ET recombineering

Correct integration of the Operon 2 to create the plasmid pSKt2PKS was confirmed via restriction analysis followed by agarose gel electrophoresis after retransformation of the Red/ET clones into E. coli DH10ß as described in the protocol.

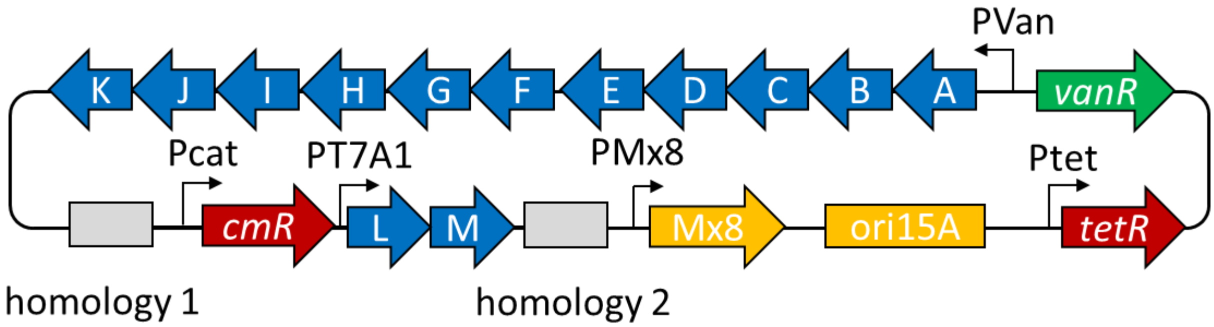

Figure S 8. Schematic depiction pSKt2PKS

The plasmid is sequenced by illumina sequencing technique to spot any errors. As we did not observe any relevant mutations in the plasmid vector, we transformed it into *M. xanthus* DK1622 by electroporation.

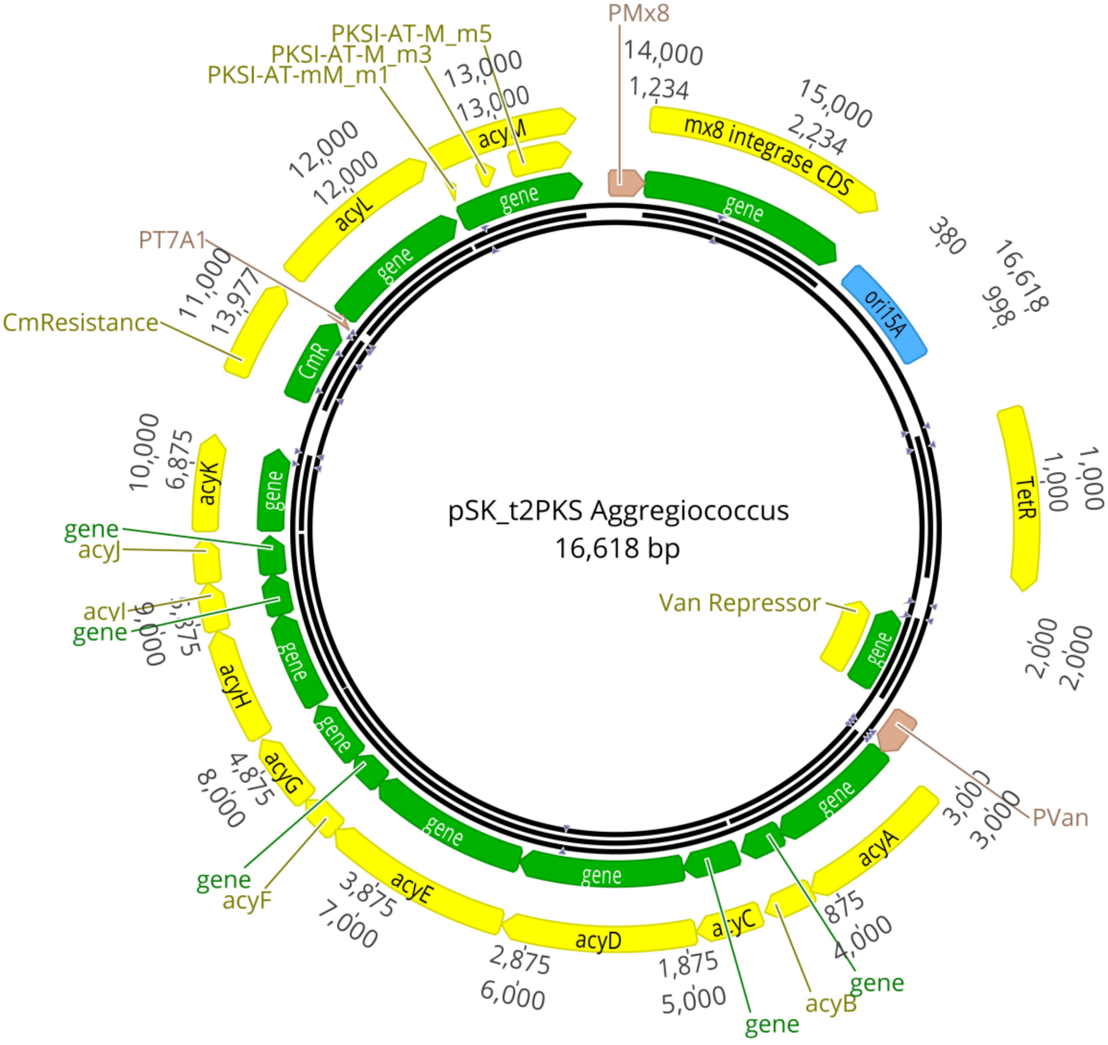

Figure S 9. Graphic depiction of the pSkt2PKS expression vector for the heterologous expression of the Aggregicyclins

4.5 Transformation of *Myxococcus xanthus DK1622*

Transformation and Mx8 based integration into the *M. xanthus* DK1622 genome are well established. Good transformation efficiency was obtained using the following electroporation protocol adapted from Hug et al..(Hug *et al.*, 2019)

4.5.1 Transformation protocol for *M. xanthus* DK1622

1) Centrifuge 2 mL of *M. xanthus* DK1622 culture in CTT Medium at OD_{600} of approx. 0.8 at 8000 rpm for 2 minutes with an Eppendorf tube table centrifuge.
2) Wash the residual cell pellet 2 times with 1 mL autoclaved ddH_2O and discard the supernatant.
3) Resuspend cells in 50 µL of ddH_2O, add 5 to 10 µL of plasmid solution (prepared from E. coli with Thermo Scientific Miniprep Kit) at a conc. of 0.3-0.4 ng/µL and transfer the solution into an electroporation cuvette.
4) Electroporation at 675 V, 400 Ω, 25 µF and 1 mm cuvette length settings for optimum electroporation efficiency.
5) Flush out the cells with 1 mL fresh CTT medium and transfer the cell suspension into a 2 mL Eppendorf tube.
6) Incubate the cells for 5 h on a shaker thermostatic to 37 °C at 300 rpm.
7) Plate the cells on Oxytet12 CTT agar after mixing the cell suspension with 3 mL of Oxytet12 CTT soft agar.
8) Yellow, spherical clones appear in the soft agar layer after 9-14 days in the 30 °C incubator.

The pSKt2PKS cluster's *attP* site integrates into the *attB* site of the myxobacterial Mx8 phage attachment site in the chromosome of *M. xanthus* DK1622 thereby inactivating the Mx8 integrase gene. (Orndorff *et al.*, 1983; Magrini *et al.*, 1999) *M. xanthus* DK1622 clones containing the pSKt2PKS plasmid integrated into the MX8 attB site of their bacterial chromosome were selected by PCR with the primers pSKt2PKS int fwd and pSKt2PKS int rev. Correct clones show a 929 bp PCR product that comprises parts of *acyD* and *acyE* and thus reveal integration of the correct plasmid.

5 Isolation and structure elucidation of the aggregicyclins

5.1 Purification of Aggregicyclins by HPLC

Purification of aggregicyclin and oxyaggregicyclin is performed using a Dionex Ultimate 3000 SDLC low pressure gradient system on a Waters Acquity CSH C18 250x10mm 5µm column with the eluents H_2O + 10 mM ammonium formate pH 7 as A and MeOH + 10 mM ammonium formate pH 7 as B, a flow rate of 5 mL/min and a column thermostatic at 30 °C. For method optimization, aggregicyclins are detected by their characteristic UV absorption at 380 nm for aggregicyclin and 470 nm for oxyaggregicyclin. Due to the aggregicyclins inherent light sensitivity, purification is done while the DAD is shut off by time dependent fraction collection. Aggregicyclin is detected in a Thermo scientific ISQ EC single quadrupole mass spectrometer for liquid chromatography tuned to the MS signals at 371.09 $[M+H]^+$ for aggregicyclin and 387.08 $[M+H]^+$for oxyaggregicyclin. Gradient is stared at 60% B, that is kept for 3 minutes followed by a ramp to 100% B during 12 minutes and a plateau at 100% B for column washing. The gradient is raped back to 60% B and reequilibrated for 2 minutes before the next injection. Aggregicyclin and oxyaggregicyclin are collected according to the appearance of their $[M+H]^+$ MS signal.

To further purify the aggregicyclin derivatives, a second semipreparative HPLC step is done using a Dionex Ultimate 3000 SDLC low pressure gradient system on a Waters XSelect CSH Phenyl-Hexyl 250x10mm 5µm column with the eluents H_2O 10 mM ammonium formate pH 7 as A and MeOH + 10 mM ammonium formate pH 7 as B, a flow rate of 5 mL/min and a column thermostatic at 30 °C. Separation is started with a plateau at 60% B for 3 minutes followed by a ramp to 100% B during 22 minutes and a plateau at 100% B during 2 minutes. The B content is ramped back to starting conditions during 30 seconds and the column is re equilibrated for 2 minutes before the next injection. The aggregicyclin and oxyaggregicyclin containing fraction are independently purified and the compound is detected by a Thermo scientific ISQ EC single quadrupole mass spectrometer for liquid chromatography tuned to the MS signals at 371.09 $[M+H]^+$ for Aggregicyclin and 387.08 $[M+H]^+$ for oxyaggregicyclin. Due to the aggregicyclins inherent light sensitivity, purification is done while the DAD is shut off by time dependent fraction collection to avoid UV based degradation of the compounds. After evaporation of the residual solvent and removal of ammonium formate by lyophilization, aggregicyclin is obtained as blackish-yellowish amorphous solids while oxyaggregicyclin is obtained as a crimson red amorphous solid. After purification, LC-*hr*MS analysis shows a single peak with an exact mass of 387.086 $[M+H]^+$ for oxyaggregicyclin and a mixture of the aggregicyclin isomers in equilibrium with an exact mass of 371.090 $[M+H]^+$. Both compounds are measured with formic acid in the eluents to be consistent with the rest of our compound library, which significantly distorts the peak shape in both UV and MS.

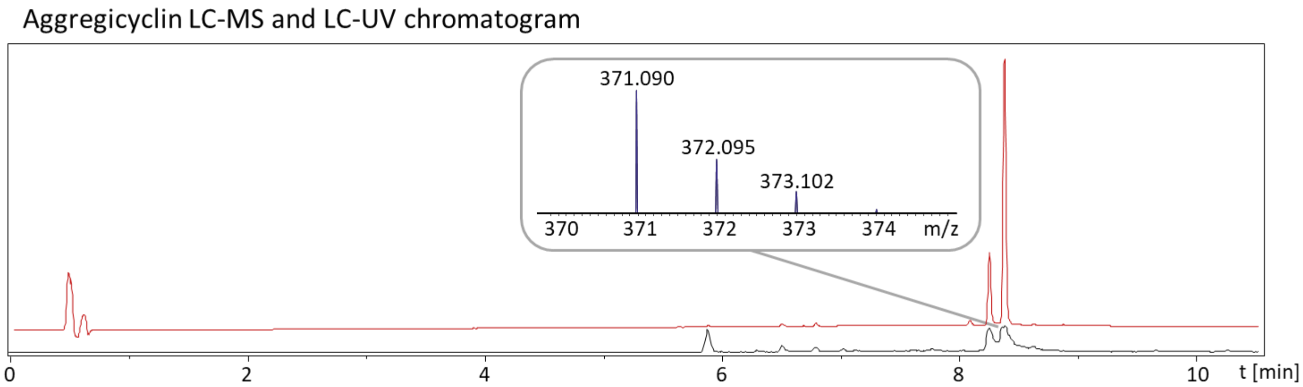

Figure S 10. LC-MS and LC-UV Chromatogram of the purified aggregicyclin isomers acquired on our uHPLC-MS platform.

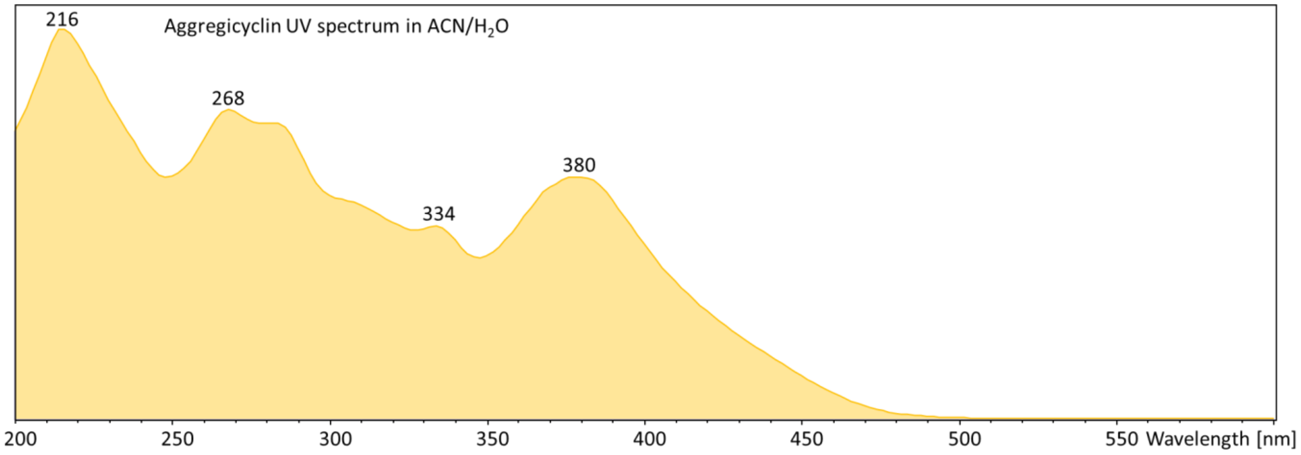

Figure S 11. UV Spectrum of aggregicyclin acquired in acetonitrile/water +0.1% FA (both main peaks show quasi identical UV spectra)

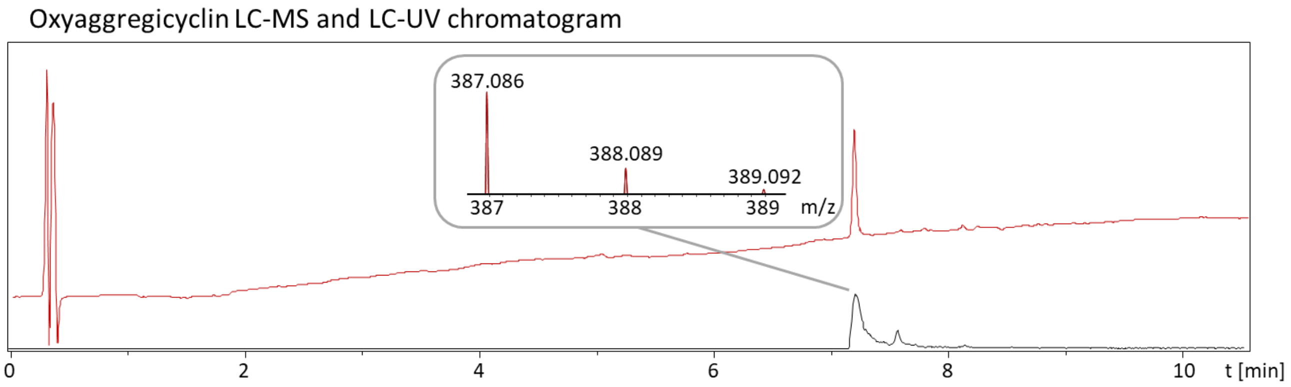

Figure S 12. LC-MS and LC-UV Chromatogram of the purified oxyaggregicyclin acquired on our uHPLC-MS platform

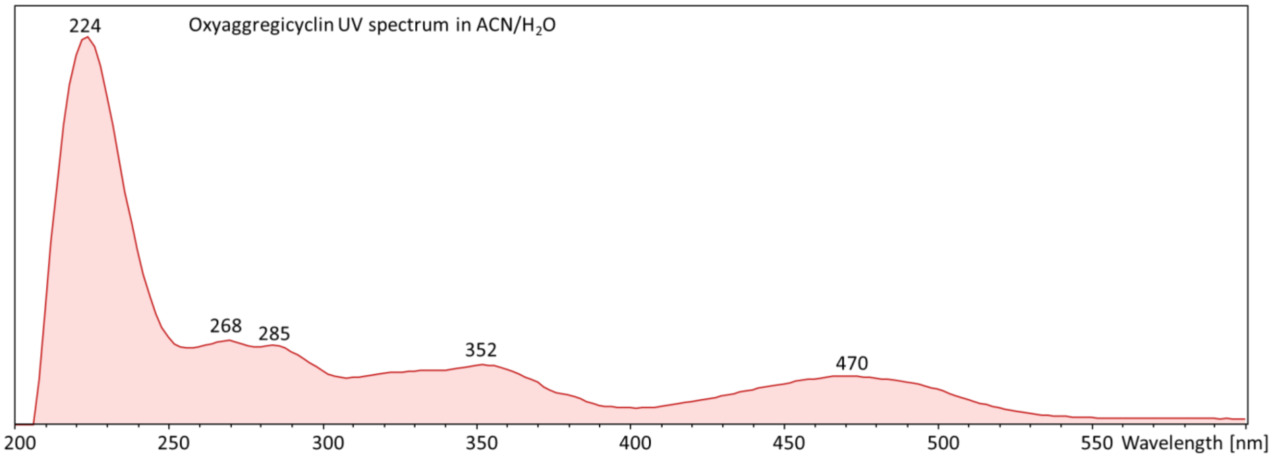

Figure S 13. UV Spectrum of oxyaggregicyclin acquired in acetonitrile/water +0.1% FA

5.2 NMR based structure elucidation

HRESI-MS analysis of oxyaggregicyclin showed an $[M+H]^+$ signal at *m/z* 387.08687 (calc. 387.0863 Δ = 0.02 ppm) consistent with the sum formula of $C_{23}H_{14}O_6$ containing 17 double bond equivalents (DBEs). The ^{1}H-NMR and HSQC spectra of oxyaggregicyclin revealed seven signals characteristic for aromatic double bonds at δ^1H 10.27 (1H, s) δC 122.9, δH 8.34 (1H, s) δC 121.5, δH 7.85 (1H, s) δC 130.7, H 7.32 (1H, s) δC 121.1, δH 7.28 (1H, s) δC 109.2, δH 7.00 (1H, s) δC 116.0 and δH 6.58 (1H, s) δC 108.3 ppm. Only one signal characteristic for a methyl group substituting an aromatic ring at δH 2.49 (3H, s) δC 21.6 was found in the HSQC spectrum. H11 and H12 are the only aromatic double bond protons showing coupling constants greater than 3 Hz, indicating them as the only aromatic protons in ortho position to each other.

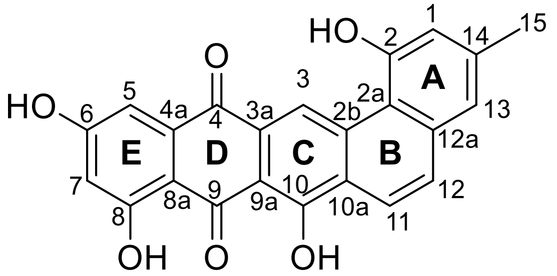

Figure S 14 Structural formula of oxyaggregicyclin with labeling and numbering used for NMR assignments.

The methyl group, which was assigned to position 15 reveals COSY correlations to the two protons in position 1 and 13, indicating them to be meta substituted to it. It furthermore shows correlations to a quaternary carbon at δC 140.3 ppm, which is based on its characteristic chemical shift and HMBC correlations the methylated carbon in position 14. There are three additional quaternary carbons correlating to H1; one at δC 118.8, one at 136.3 and one at 157.8 ppm. Based on their characteristic chemical shifts, the two carbons at δC 118.8 and 136.3 ppm are the bridgehead atoms connecting aromatic ring A with B. The chemical shift of the de-shielded carbon 2 at 157.8 ppm indicates its substitution with a hydroxyl function. Based on this assignment, 2a is its neighboring carbon at δC 118.8 ppm and the carbon at δC 136.3 ppm was assigned as 12a. C2 only shows correlations to H1 and not H13 indicating it – alongside the more shielded shifts of H1 and C1 compared to H13 and C13—in meta position to 1. It is noteworthy that the ^4J correlation between H1 and C12a is only detectable with a constant 13 of 8 Hz.

There is a strong HMBC correlations between H13 and C12 which hints towards their ^3J coupling, further supported by a very week COSY correlation between the respective protons. 12 was therefore assigned to the B-ring. As already mentioned before H11 was identified to be in meta position to H12 showing a coupling constant of 9 Hz. Additionally, to their correlations with atoms participating in the A-ring, they reveal correlations to two quaternary carbons which are not correlating to any of the protons allocated in the A-ring. Their characteristic chemical shift at δC 137.5 and 126.9 ppm indicate them as bridgehead atoms 2b and 10a between the B and C ring. H11 shows correlations to the more deshielded carbon at δC 137.5 ppm, which based on its characteristic chemical shift is in meta position to another aromatic proton,

so this carbon was assigned as C2b. H12 shows correlations with the more shielded carbon at δC 126.9 ppm, so this carbon was assigned as 10a. The ^2J coupling of H11 and 10a is not detectable in neither of the HMBC experiments. These correlations often lie under the limit of detection in polyaromatic natural products as already observed for chelocardin-derivatives exemplarely.(Lukežič *et al.*, 2019) H11 reveals further correlations to a deshielded quaternary carbon at δC 161.3 ppm, which was assigned as C10 bearing a hydroxyl group.

The quaternary carbon 10a shows correlations to the aromatic proton at δH 10.27 ppm, which was assigned to position 3. Besides correlations to atoms participating in the B-ring, this proton reveals correlations to two more quaternary carbons at δC 127.4 and 110.5 ppm. The correlation to the carbon at δC 110.5 ppm is very strong, whereas the correlation to the carbon at δC 127.4 ppm is only visible with a constant 13 of 8 Hz. We therefore assigned the carbon at δC 127.4 ppm as 3a and the carbon at δC 127.4 ppm as 9a, forming the bridgehead atoms connecting the C- and D-ring. H3 furthermore shows correlations to two highly deshielded carbons at δC 181.7 and 182.0 ppm, which were assigned as ketones based on their characteristic chemical shift.

There are two further protons detectable in the NMR spectra, both represented as singlets without any COSY correlations which reveal correlations to four quaternary carbons at δC 111.0, 137.5, 166.2 and 166.8 ppm. Based on their de-shielded chemical shift, the carbons at δC 166.2 and 166.8 ppm are hydroxyl substituted. The two quaternary carbons at δC 111.0 and 137.5 ppm show characteristic chemical shifts for bridgehead atoms, wherefore we assigned them as 8a and 4a connecting the D and E ring. H5 shows strong correlations to the carbon at δC 137.5 ppm, but only a weak correlation to the carbon at 111.0 ppm only detectable with a constant 13 of 6 Hz. This carbon does not show correlations to the second proton in the E-ring at all, so we concluded its meta position relative to H5 and para to H7 and assigned it as 4a. Based on the NMR data, there were two substitution patterns possible in the E ring, as the chinone system in ring D interrupt all NMR correlations between atoms participating in the C and E ring. Our investigation of the biosynthetic formation of oxyaggregicyclin however allowed us to confirm the substitution pattern shown in figure S15 on the left-hand side.

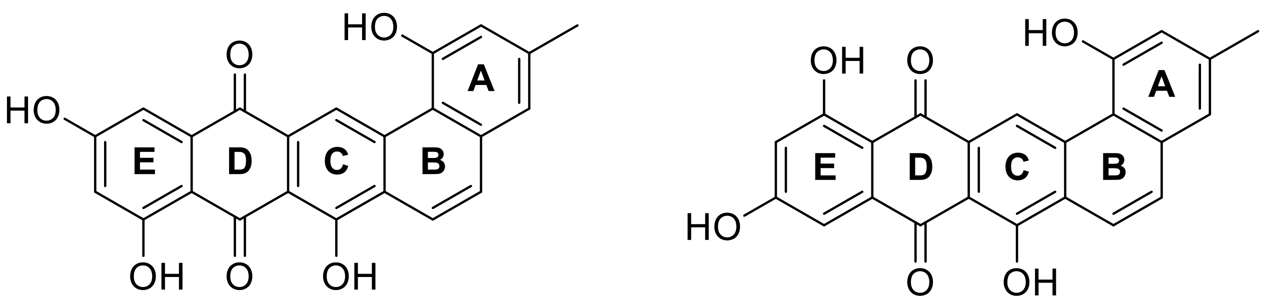

Figure S 15. Two possible substitution pattern of the E-ring based on the NMR data. Biosynthesis investigation allowed us to confirm the left structure to be the correct one based on type-II PKS biosynthesis logic.

HRESI-MS analysis of aggregicyclin showed an $[M+H]^+$ signal at *m/z* 371.09206 (calc. 371.09140 Δ = 1.78 ppm) consistent with the sum formula of $C_{23}H_{14}O_5$ containing 17 double bond equivalents (DBEs). The ^{1}H-NMR and HSQC spectra of aggregicyclin comparable to oxyaggregicyclin revealed seven signals characteristic for aromatic double bonds at δH 9.26 (1H, s) δC 120.3, δH 8.16 (1H, s) δC 121.5, δH 7.58 (1H, s) δC 126.9, δH 7.20 (1H, s) δC 120.7, δH 6.97 (1H, s) δC 115.0, δH 6.16 (1H, s) δC 102.3 and δH 5.76 (1H, s) δC 109.0 ppm and one signal characteristic for a methyl group substituting an aromatic ring at δH 2.46 (3H, s) δC 21.4 ppm. Additionally, we detect a methine group at δH 4.74 (3H, s) δC 58.9 ppm lacking in oxyaggregicyclin.

Noteworthy there is a second data set with 1/3 intensity detectable compared to the primary dataset, so we called the two components aggregicyclin A and B (see figure S16). In the B dataset three HSQC signals correlating to aromatic protons are shifted to δH 8.94 (1H, s) δC 120.0, δH 6.82 (1H, s) δC 114.8 and δH 6.10 (1H, s) δC 109.4 ppm.

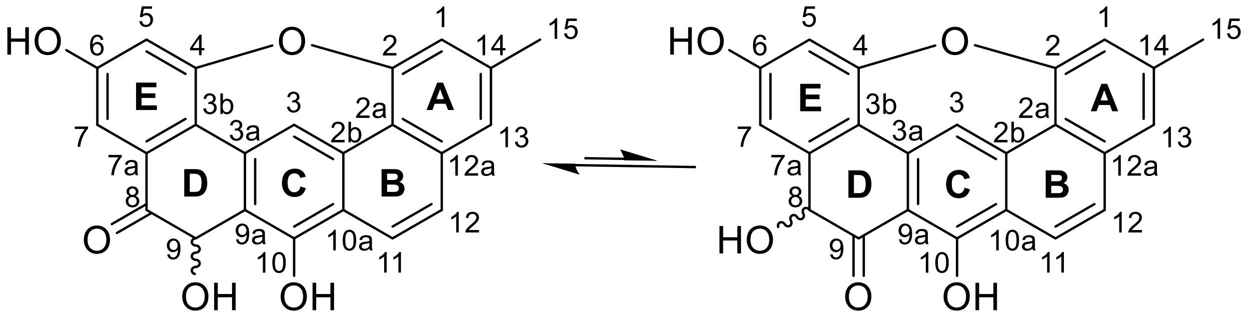

Aggregicyclin A   Aggregicyclin B

Figure S 16 Structural formula of aggregicyclin with labeling and numbering used for NMR assignments.

Comparable to oxyaggregicylin the methyl group 15 reveals correlations to two of the aromatic double bond protons and a quaternary carbon at 139.0 ppm. The two aromatic double bond protons 1 and 13 reveal HMBC correlations to three additional quaternary carbons at δC 157.9, 117.7 and 136.2 ppm. Based on their highly similar chemical shifts and same correlations observable compared to oxyaggregicyclin, we concluded that the A ring shows the same substitution pattern. Also, in the B ring we could assign two aromatic protons, which show correlations to the two quaternary carbons C2b and C10a forming the bridgehead atoms connecting the A and B ring, as well as two further quaternary carbons at δC 121.9 and 137.4 ppm. We therefore also assigned the B ring as presenting the same substitution pattern compared to oxyaggregicyclin. The aromatic proton H3 at δC 9.26 (1H, s) δC 120.3 ppm reveals correlations to the two bridgehead atoms 3a and 9a connecting the B and C ring, as well as to three additional quaternary carbons at δC 159.9, 111.8 and 145.6 ppm. The C ring therefore also displays the same substitution pattern compared to oxyaggregicyclin. The deviations in the chemical shifts in the C ring however already indicated that the D rings differs from oxyaggregicyclin.

The deshielded quaternary carbon at δC 159.9 ppm, which was assigned as C10, reveals HMBC correlations to the methine group at δH 4.74 ppm, the bridgehead atom 3a, as well as C3, so it was assigned

to the D ring. The methine group in turn shows correlations to a deshielded quaternary carbon at δC 191.3 and two more shielded carbons at δC 137.1 and 145.6 ppm. Based on their characteristic chemical shifts, the carbon at δC 191.3 ppm was assigned as ketone in position 8 and the carbons at δC 137.1 and 145.6 ppm as bridgehead atoms 3b and 7a connecting the D and E ring.

Based on the strong correlation between the methine and the keto group, we assigned them in ortho position. This hypothesis is further supported by the finding that we detect three isomers in our UHPLC-MS analysis, which likely correspond to the keto-enol tautomers of aggregicyclin. This equilibrium is dependent on the pH of the aggregicyclin solution, which is why we detect more of the earlier eluting peak corresponding to aggregicyclin B in the crude extract at neutral pH, but more of the later eluting peak corresponding to aggregicyclin A in the NMR sample under acidic conditions. Under acidic conditions we also observe that the keto-enol tautomerism seem to happen faster as represented by only detecting the enol in the crude extract.

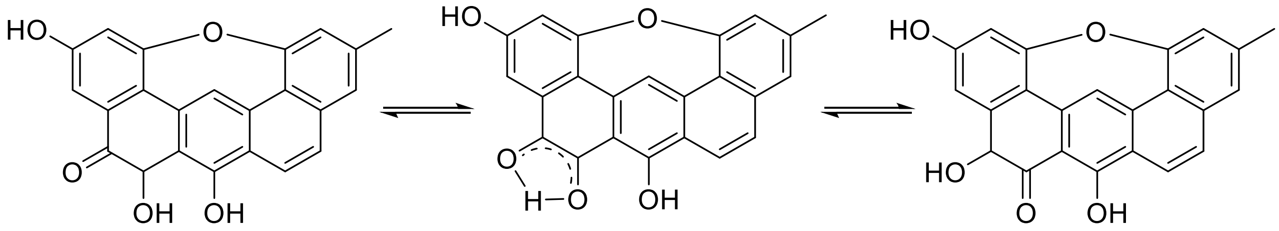

Figure S 17 Keto-enol tautomerism of aggregicyclin in solution.

In the E ring we observe two aromatic protons, which show correlations two deshielded quaternary carbons at δC 165.8 and 165.0 ppm, besides correlations to the two bridgehead atoms 3b and 7a. The aromatic proton at δH 5.76 ppm was assigned as H7, as it shows strong correlations to the keto function in position 8 of aggregicyclin A. In aggregicyclin B this proton is shifted to δH 6.10 ppm and shows correlations to the methine group in position 8 of aggregicyclin B, which is why we assumed this correlation to be a ^3J correlation. The two aromatic signals in position 5 and 7 show a coupling constant of 2.1 Hz in aggregicyclin A and of 1.7 Hz in aggregicyclin B, so they were assigned in meta position.

We detect three exchangeable protons at 12.92, 12.03 and 7.93 ppm. The proton at 12.92 ppm shows correlations to C9a, 10 and 10a indicating it as C10 OH. As the hydroxyl function at the methine in position 9 (or 8 respectively for the B derivative) undergoes the observed keto-enol tautomerization, we could not detect any correlations of the proton at 7.93 ppm, which we assigned based on its characteristic chemical shift as a secondary hydroxyl function. Unfortunately, also for the proton at 12.03 ppm displays only weak correlations hardly above the signal to noise ratio. The aggregicyclin sum formula clearly indicates that two remaining hydroxyl functions must be connected *via* an ether bridge, yet we didn't observe any correlations to pinpoint its exact position. The hydroxyl proton at δH 12.92 ppm however, strongly supports the aggregicyclin structure depicted in figure S16: It exhibits HMBC correlations to three quaternary carbons, of which one was assigned as hydroxyl-bearing carbon based on its characteristic chemical shift of δC 195.9 ppm. The other two were assigned as the neighboring quaternary carbons based on their correlations to the protons in ring B and D, suggesting this proton to be carried by the hydroxyl function in ring C. The

positioning of a free hydroxyl function in the C-ring is additionally supported by the deshielded shift of the respective proton at *δ*H 12.92 ppm, which better fits the predicted chemical shift of a proton in this position at *δ*H 11.30 ppm (Figure S18 **2**) in comparison to a free hydroxyl function in the A ring 9.04 ppm (Figure S18 **1**) and resembles the deshielding effect of neighboring ketones in aromatic polyketides as described for tiancimycin exemplarily.

To confirm this hypthesis, we tried to visualize long range HMBC correlations. We performed HMBC experiments with varying constant 13 values (6, 8 and 10 Hz) and changed the NMR solvent to deuterated methanol, but both all our attempts failed to provide the required information. Due to the very poor solubility and instability of aggregicyclin in most chemical solvents, we were unable to acquire NMR data in different solvents and CIGAR-HBMC-experiments were also unsuccessful, most likely due to its comparably low sensitivity.(Araya-Maturana *et al.*, 2008)

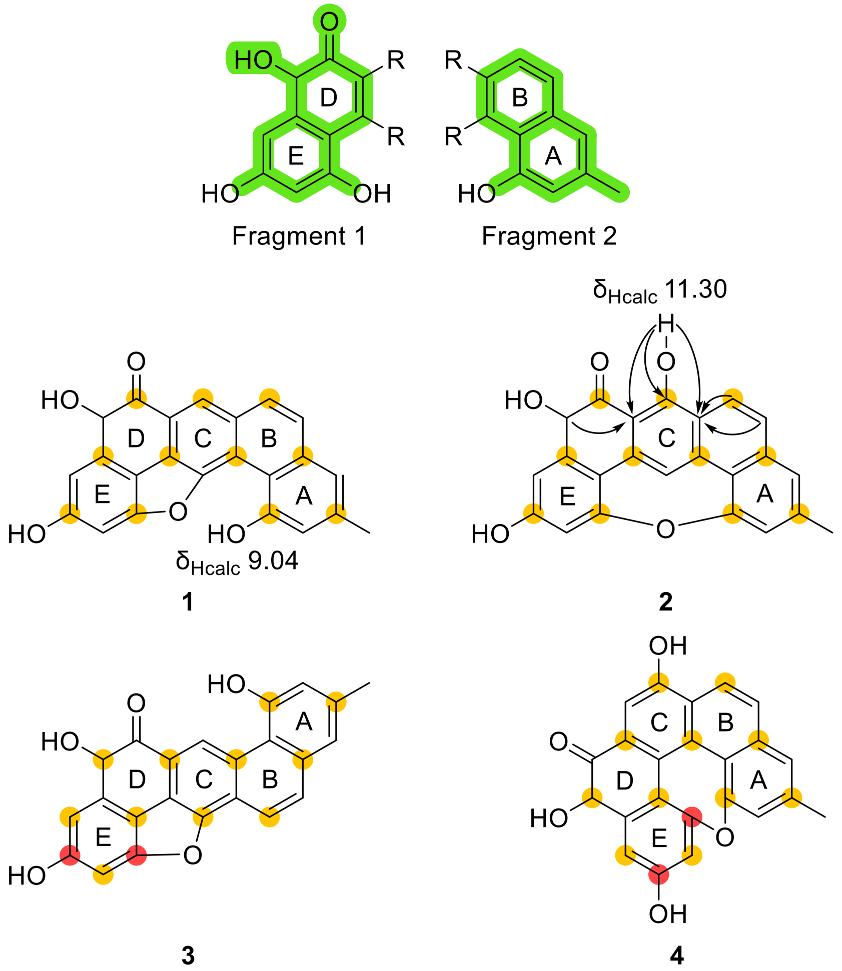

Figure S 18 Possible structures of aggregicyclin (**1-4**) based on the two key fragments **1** and **2** that could unambiguously (green) be assigned from the NMR spectra. Key COSY-correlations that led to the proposed structure **2** as most likely aggregicyclin structure shown as arrows. Predicted hydroxylation pattern based on incorporated acetate units marked in yellow, diverging hydroxylations in red.

Table S 8. NMR spectroscopic data for oxyaggregicyclin.

Position	NMR data in THF-d_8			
	δ_C	δ_H (J in Hz)	COSY correlations	HMBC correlations
1	116.0	7.00, bs	13, 15	2, 2a, 13, 15
2	157.8		-	-
2a	118.8	-	-	-
2b	137.5	-	-	-
3	122.9	10.27, bs	-	2a, 4, 9a, 10a, 11, 12a
3a	127.4	-	-	-
4	181.7	-	-	-
4a	137.5	-	-	-
5	109.2	7.28, bs	6	4a, 6, 7, 8a, 9
6	166.8	-	-	-
7	108.3	6.58, bd (2.2)	8	5, 6, 8, 8a
8	166.2	-	-	-
8a	111.0	-	-	-
9	182.0	-	-	-
9a	110.5	-	-	-
10	161.3	-	-	-
10a	126.9	-	-	-
11	121.5	8.34, d (9.0)	12	2b, 10, 12a
12	130.7	7.85, d (9.0)	11	2a, 2b, 10a, 13
12a	136.3	-	-	-
13	121.1	7.32, bs	1, 15	1, 2a, 2b, 12, 15
14	140.3	-	-	-
15	21.3	2.49, s	1, 13	1, 13, 14
2-OH	-	10.83, s	-	n.d.
5-OH	-	9.85, bs	-	n.d.
7-OH	-	12.36, bs	-	n.d.
10-OH	-	n.d.	-	n.d.

n.d. = not detectable

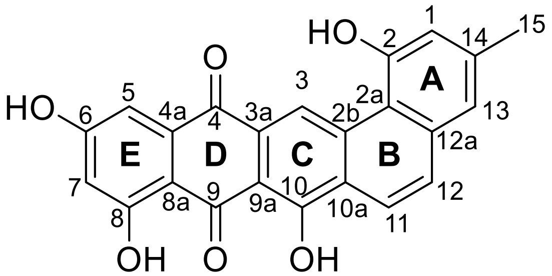

Table S 9. NMR spectroscopic data for aggregicyclin. Peaks different in B derivative marked italic.

Position	NMR data in THF-d_8			
	δ_C	δ_H (J in Hz)	COSY correlations	HMBC correlations
1	114.9	6.97, bs	13, 15	2, 2a, 12a, 13, 14, 15
1-B	*114.8*	*6.82, d (1.3)*	*13, 15*	*2, 2a, 12a, 13, 14, 15*
2	157.9	-	-	-
2a	117.7	-	-	-
2b	137.4	-	-	-
3	120.3	9.26, s	-	2, 2a, 9, 9a, 10, 10a, 12a
3-Br	*120.0*	*8.94, s*	*-*	*2, 2a, 9, 9a, 10, 10a, 12a*
3a	145.6	-	-	-
3b	110.7	-	-	-
4	165.8	-	-	-
5	102.3	6.16, d (2.1)	7	3a, 3b, 4, 6, 7, 8
6	165.0	-	-	-
7	102.3	5.76, bs	5	3b, 4, 5, 6, 8, 9
7-B	*109.4*	*6.10, d (1.7)*	*7*	*3b, 5, 6, 7a, 8, 9*
7a	137.1	-	-	-
8	191.3	-	-	-
8-B	*58.9*	*4.74, s*	*-*	*3b, 4, 6, 7 ,9, 9a*
9	58.9	4.74, s	-	3, 3a, 7a, 8, 10
9-B	*191.3*	*-*	*-*	*-*
9a	111.8	-	-	-
10	159.9	-	-	-
10a	121.9	-	-	-
11	121.5	8.16, d (9.1)	12	2a, 2b, 10, 10a, 12a
12	126.9	7.58, d (9.1)	11	2, 2a, 2b, 10a, 12a, 13
12a	136.2	-	-	-
13	120.7	7.20, bs	1, 15	1, 2, 2a, 2b, 12, 15
14	139.0	-	-	-
15	21.4	2.46, bs	1, 13	1, 13, 14
6-OH	-	12,0, bs	-	-
9-OH	-	7.93, s	-	-
10-OH	-	12.9, bs	-	9a, 10, 10a
10-OH-B	-	*12.7, bs*	-	*9a, 10, 10a*

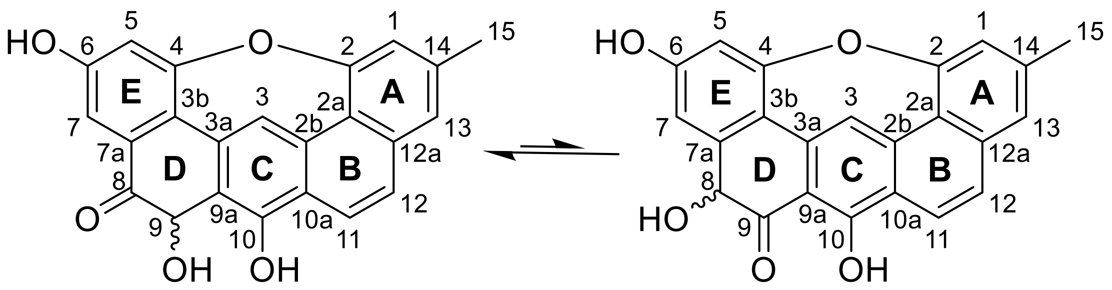

Aggregicyclin A   Aggregicyclin B

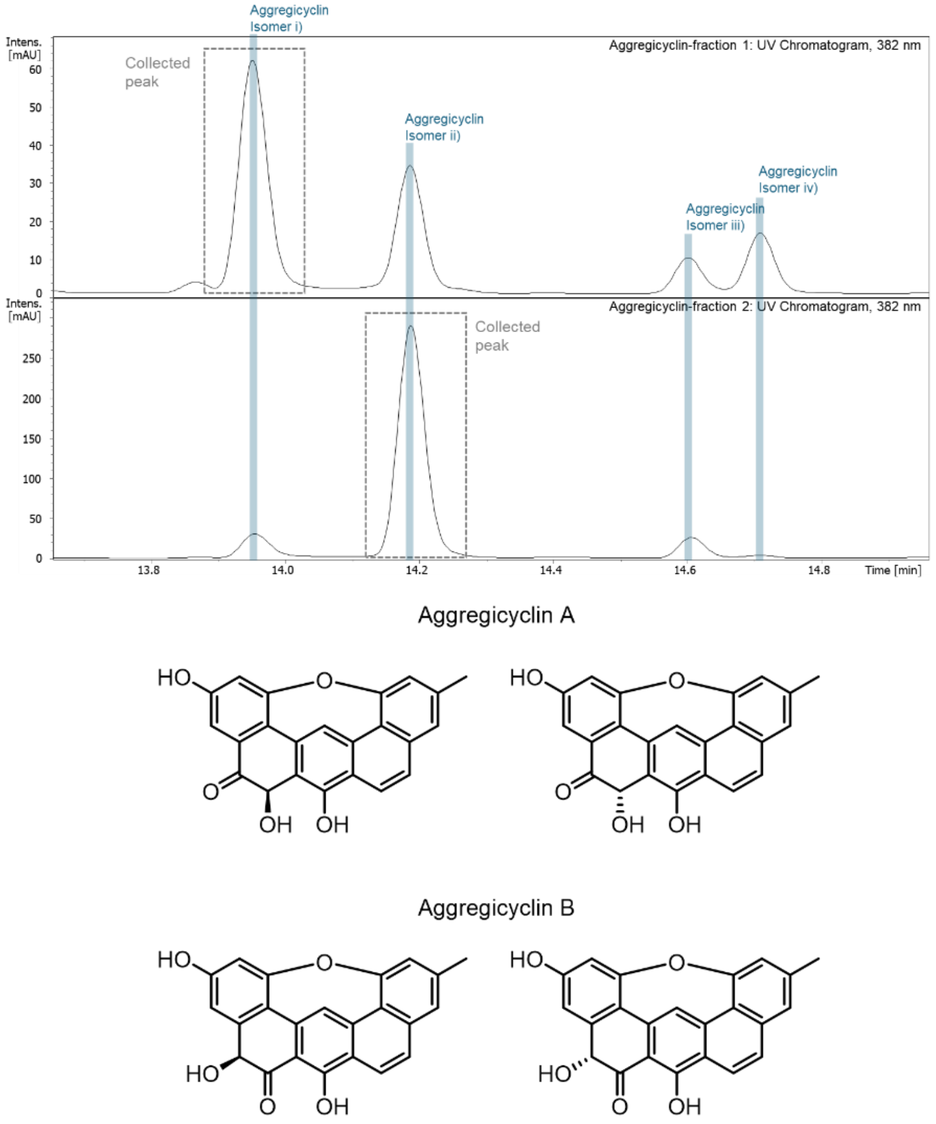

Figure S 19 Observed aggregicyclin isomers and their assignment as keto-enol-tautomers.

The NMR data of aggrigicyclin clearly support the presence of two keto–enol tautomeric forms within the molecule. In aggregicyclin A, the keto function is located at position 8, whereas in aggregicyclin B it is situated at position 9 (see Table S9). Under achiral HPLC conditions, four peaks with an identical *m/z* value of 371.09 were observed (see Figure 2). These peaks interconvert after separation (see Figure S19), indicating a dynamic equilibrium between the species. We attribute these four signals to keto–enol tautomerization, resulting in a total of four isomers: two enantiomers each of aggregicyclin A and aggregicyclin B. Although separation of enantiomers on an achiral column is unusual, this behavior can likely be explained by self-disproportionation of enantiomers (SDE) driven by π–π interactions within the conjugated aromatic system. Similar phenomena have previously been reported for other aromatic compounds, such as binaphthol.[11]

6 Biosynthesis of the aggregicyclins

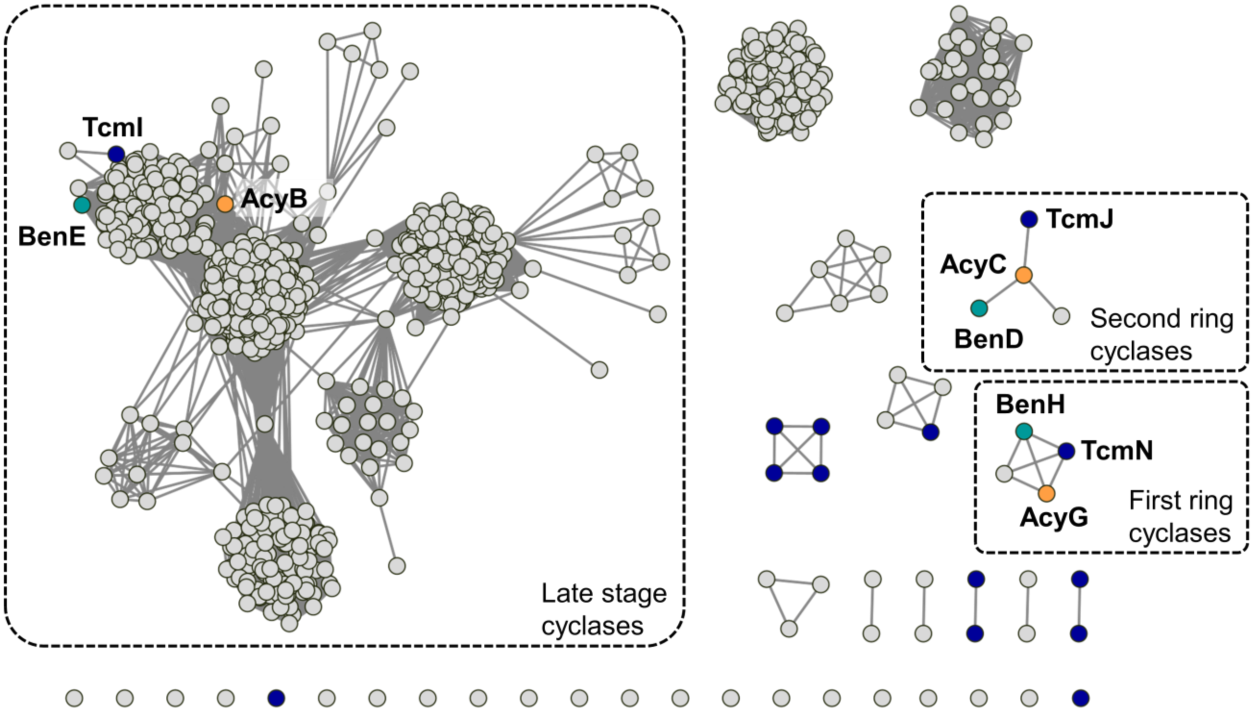

Figure S 20 Sequence similarity network of type II PKS cyclases. Sequences were retrieved from UniProt and complemented with manually added sequences from the aggregicyclin (AcyB, AcyC, and AcyG; yellow) and benastatin (BenD, BenE, and BenH; teal) pathways. Experimentally characterized cyclases from other pathways are shown in blue, with the closest homologues TcmI, TcmJ, and TcmN from tetracenomycin biosynthesis labeled.

7 NMR spectra employed in aggregicyclin structure elucidation

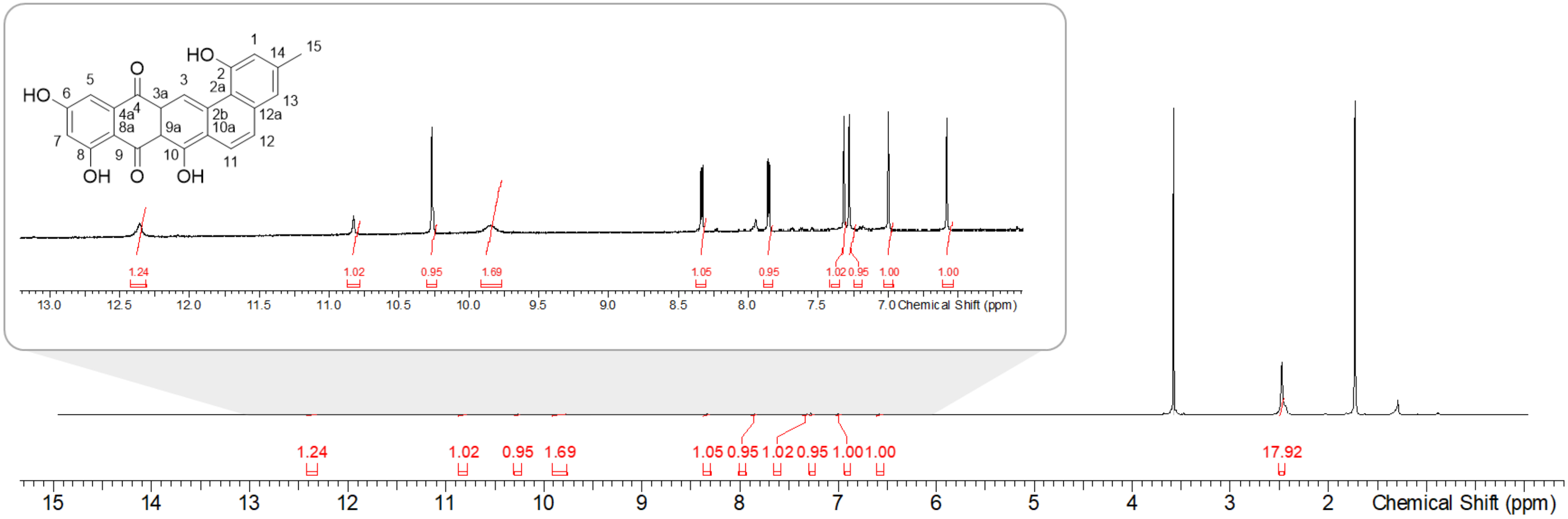

Figure S 21. ^{1}H-spectrum of oxyaggregicyclin in THF-d_8 at 700 MHz.

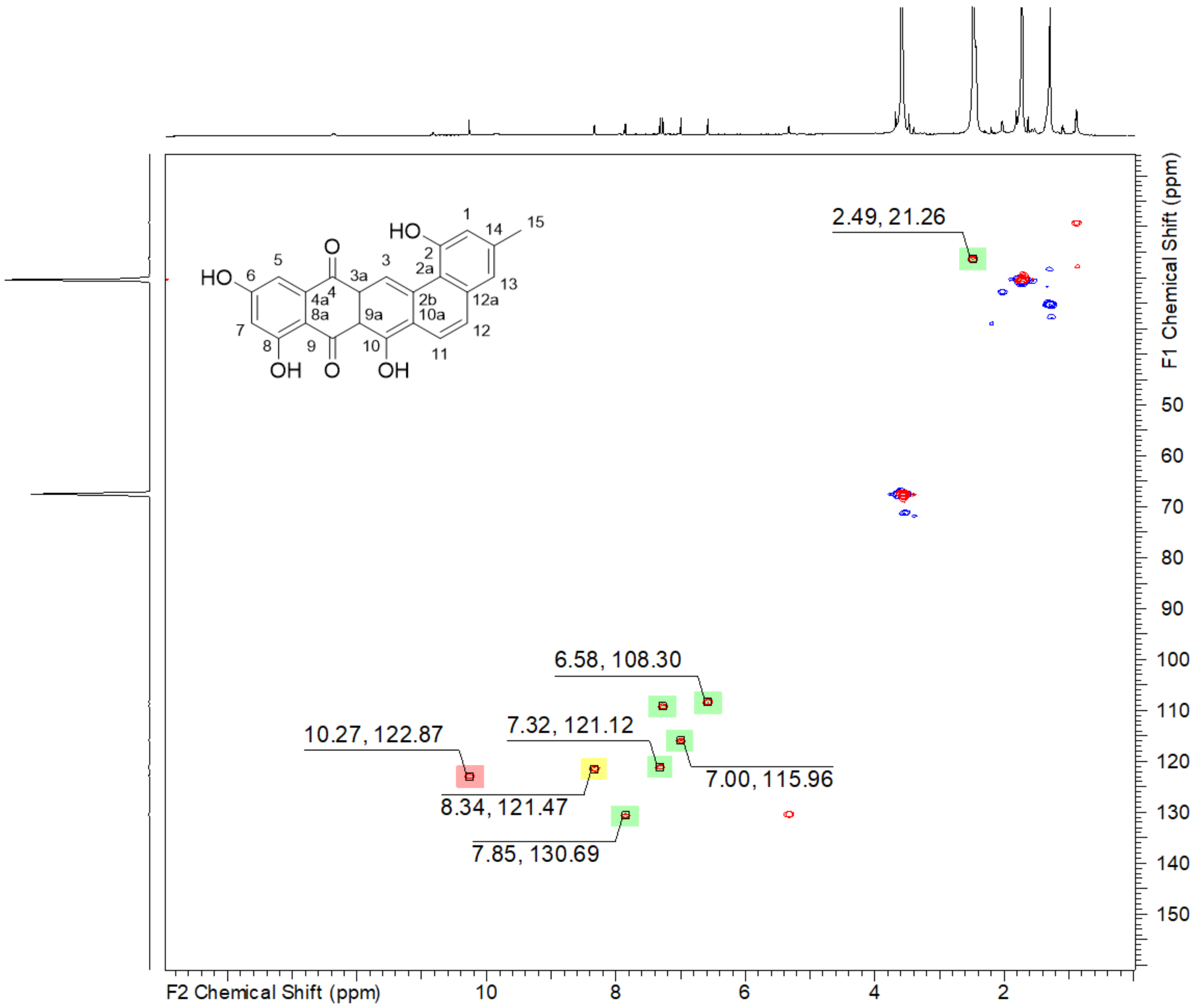

Figure S 22. HSQC-spectrum of oxyaggregicyclin in THF-d_8 at 700/175 MHz.

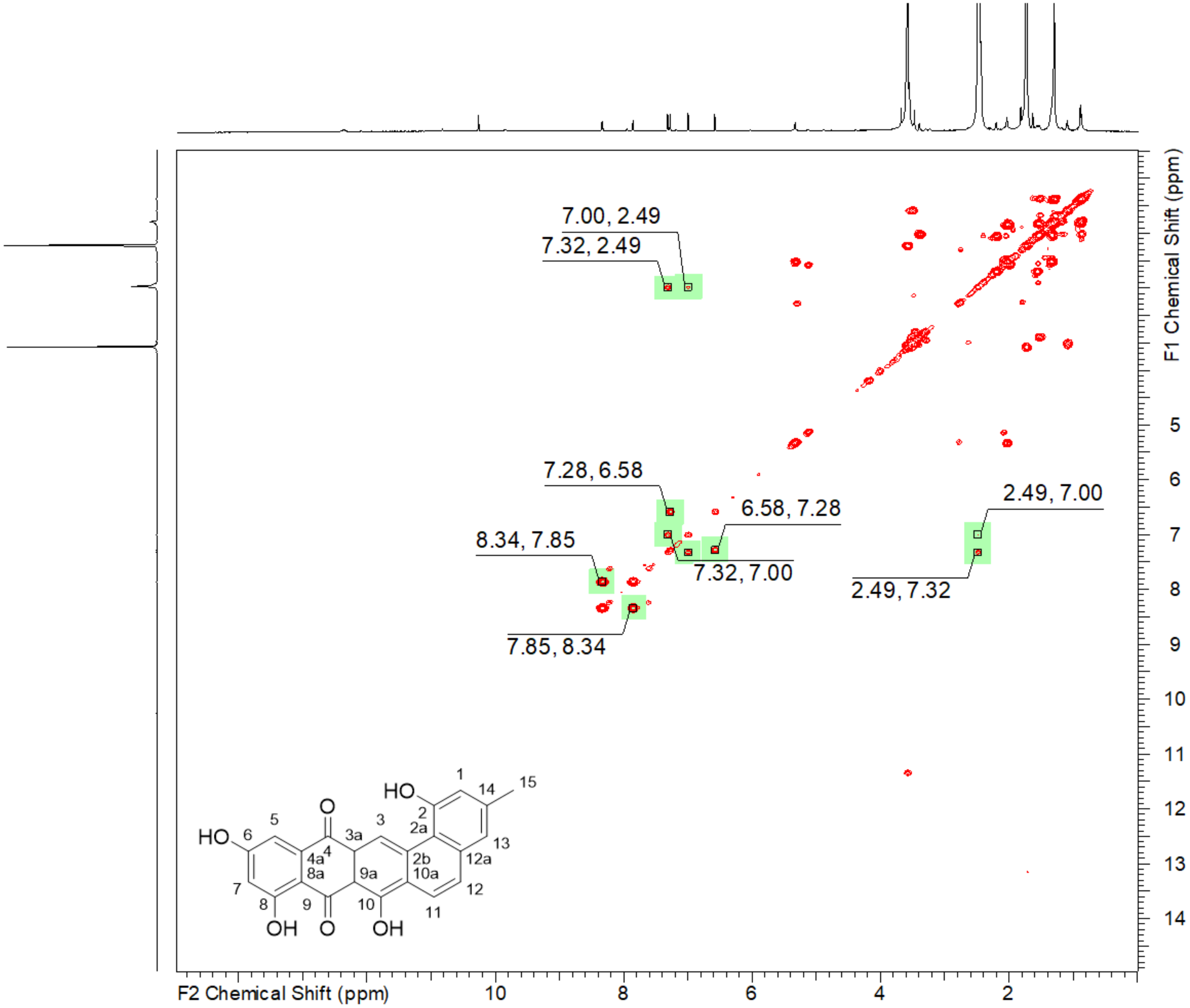

Figure S 23. COSY-spectrum of oxyaggregicyclin in THF-d_8 at 700 MHz.

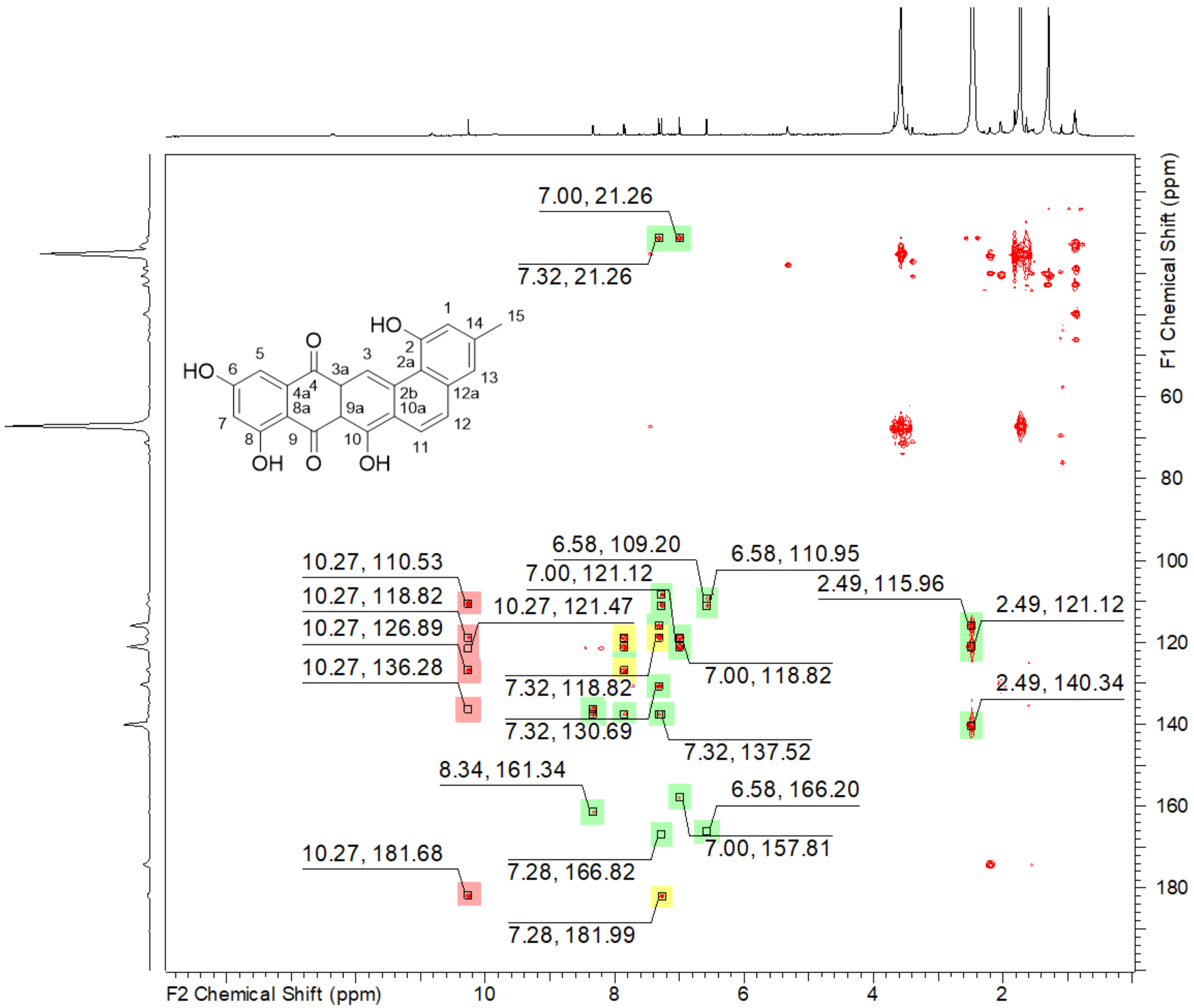

Figure S 24. HMBC-spectrum of oxyaggregicyclin in THF-d_8 at 700/175 MHz optimized for constant 13 = 6 Hz.

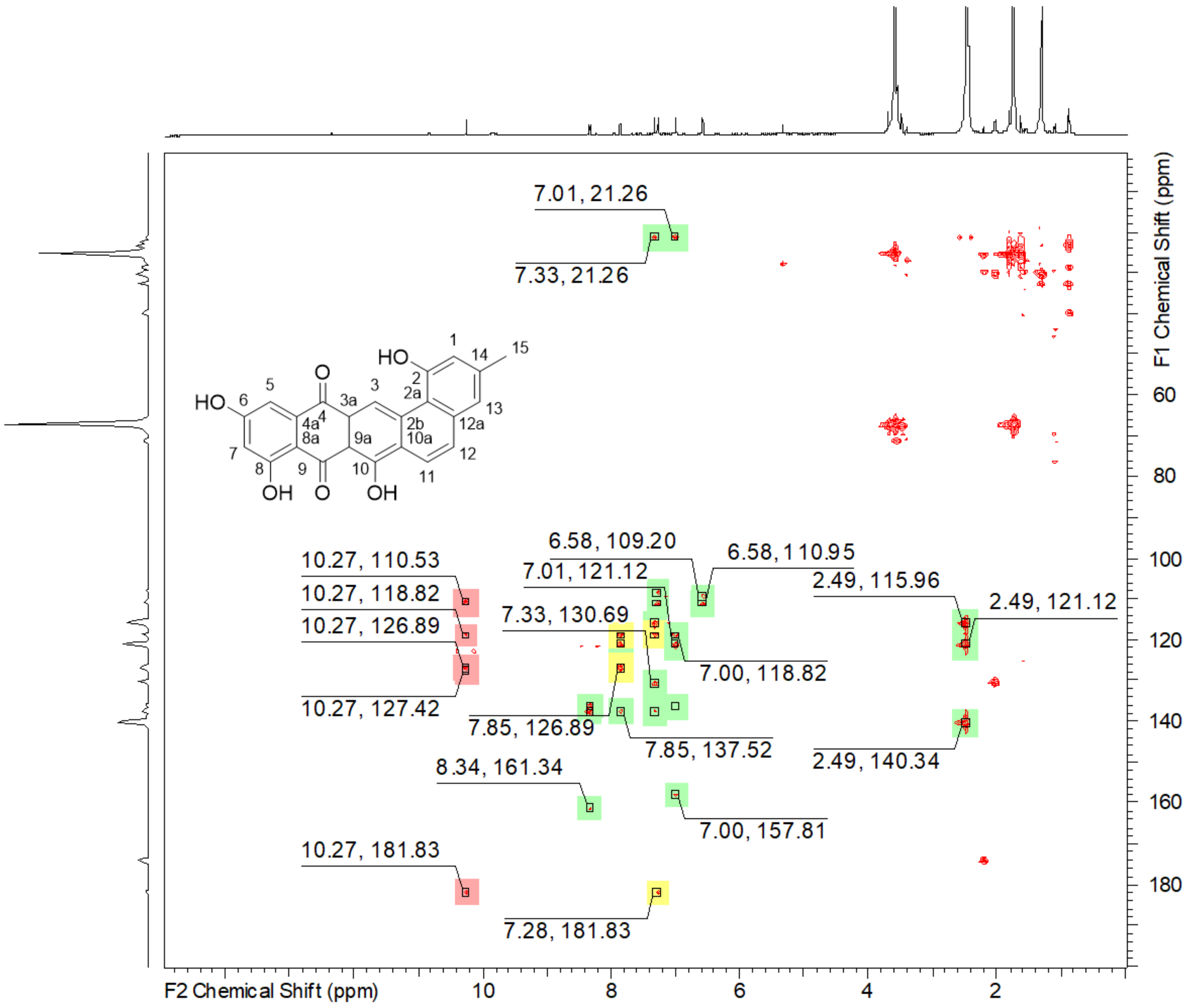

Figure S 25. HMBC-spectrum of oxyaggregicyclin in THF-d_8 at 700/175 MHz optimized for constant 13 = 10 Hz.

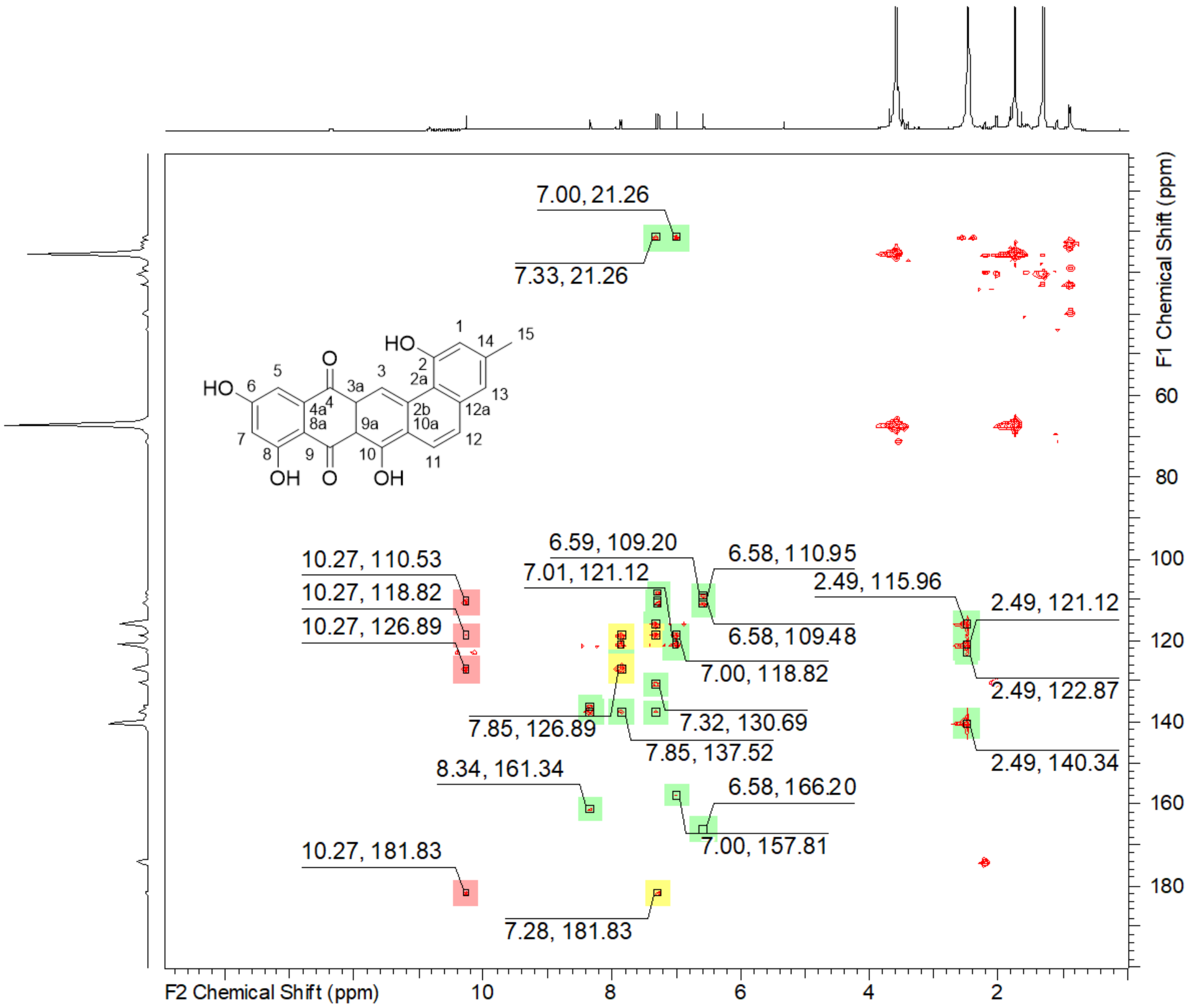

Figure S 26. HMBC-spectrum of oxyaggregicyclin in THF-d_8 at 700/175 MHz optimized for constant 13 = 8 Hz.

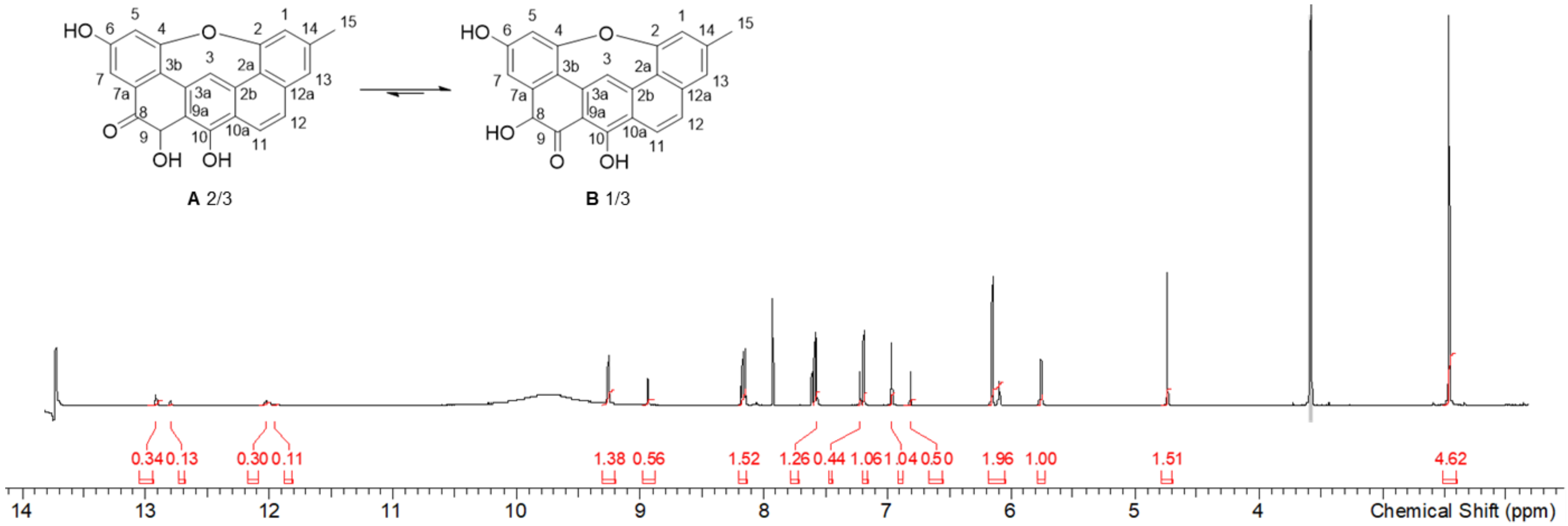

Figure S 27. ^{1}H-spectrum of aggregicyclin in THF-d_8 at 700 MHz.

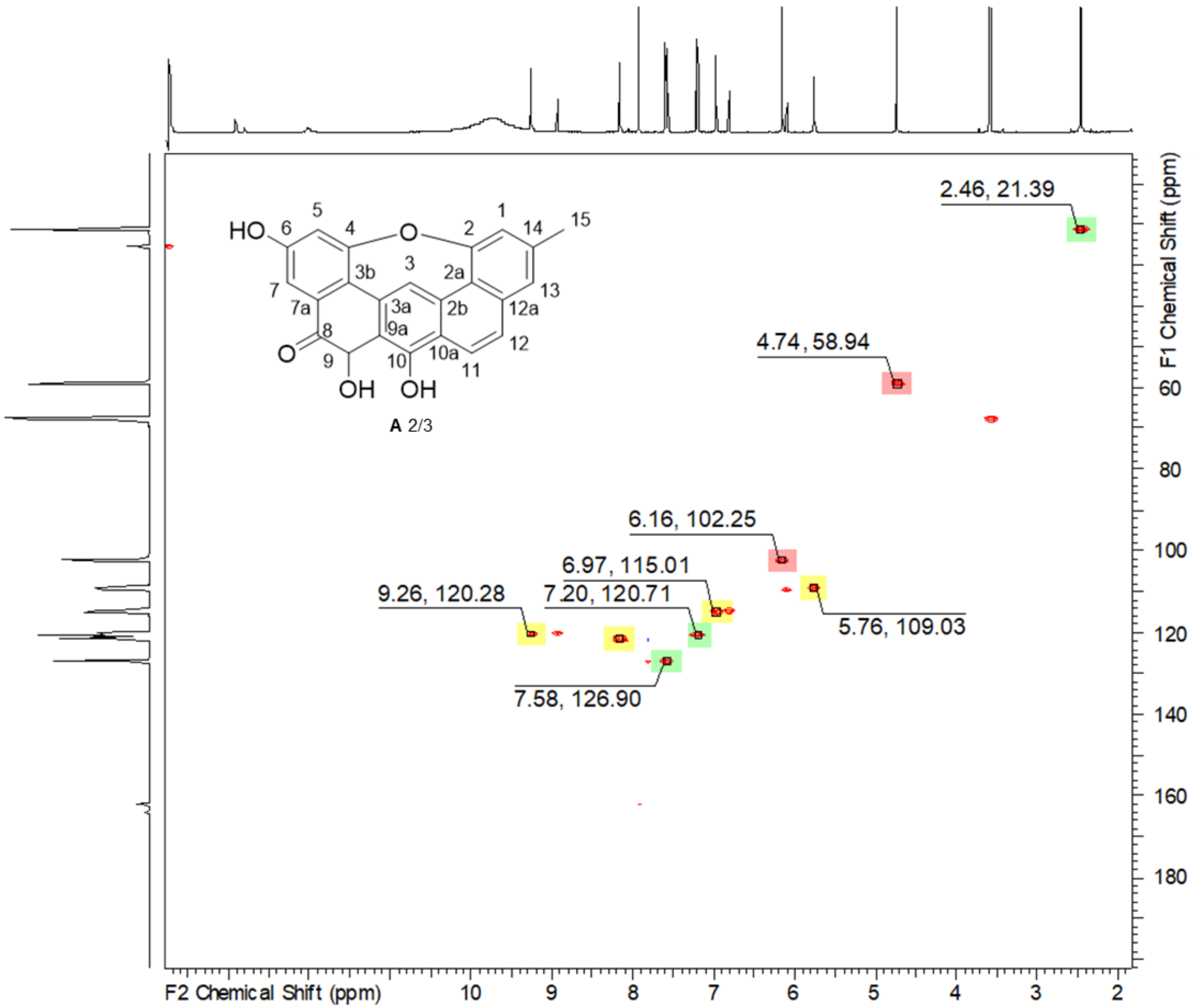

Figure S 28. HSQC-spectrum of aggregicyclin in THF-d_8 at 700/175 MHz. Peaks marked were assigned to A derivative.

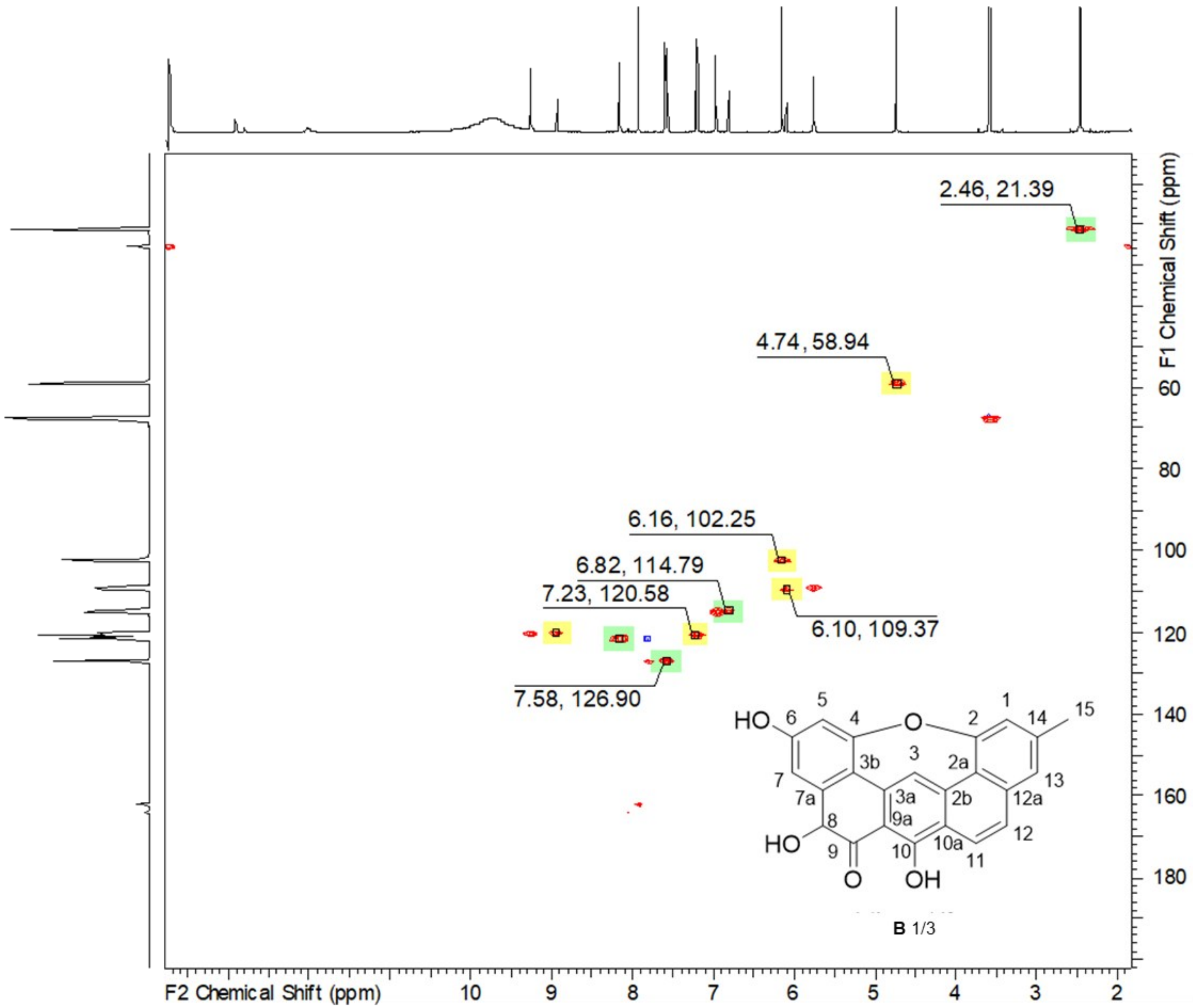

Figure S 29. HSQC-spectrum of aggregicyclin in THF-d_8 at 700/175 MHz. Peaks marked were assigned to B derivative.

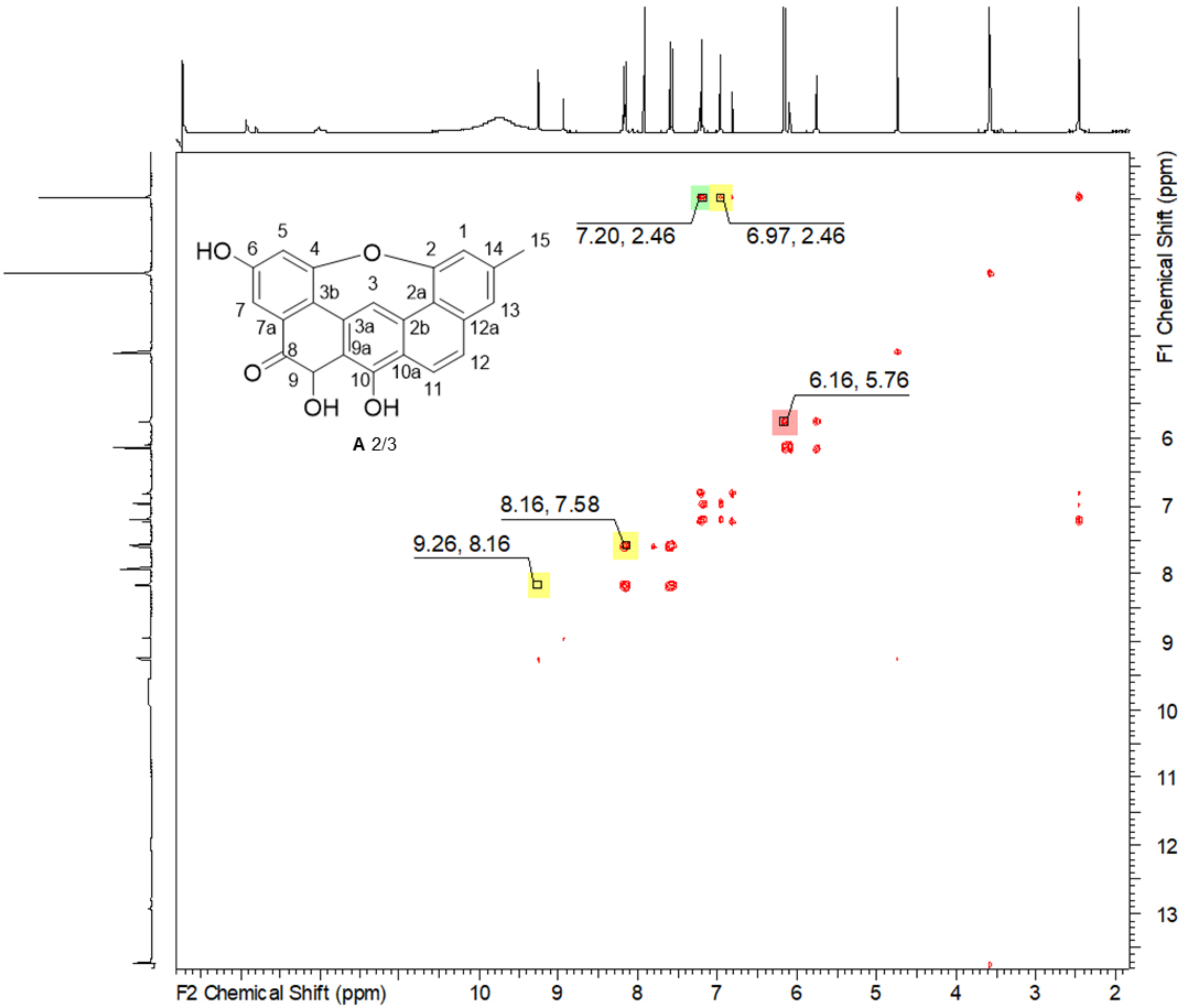

Figure S 30. COSY-spectrum of aggregicyclin in THF-d_8 at 700/175 MHz. Peaks marked were assigned to A derivative.

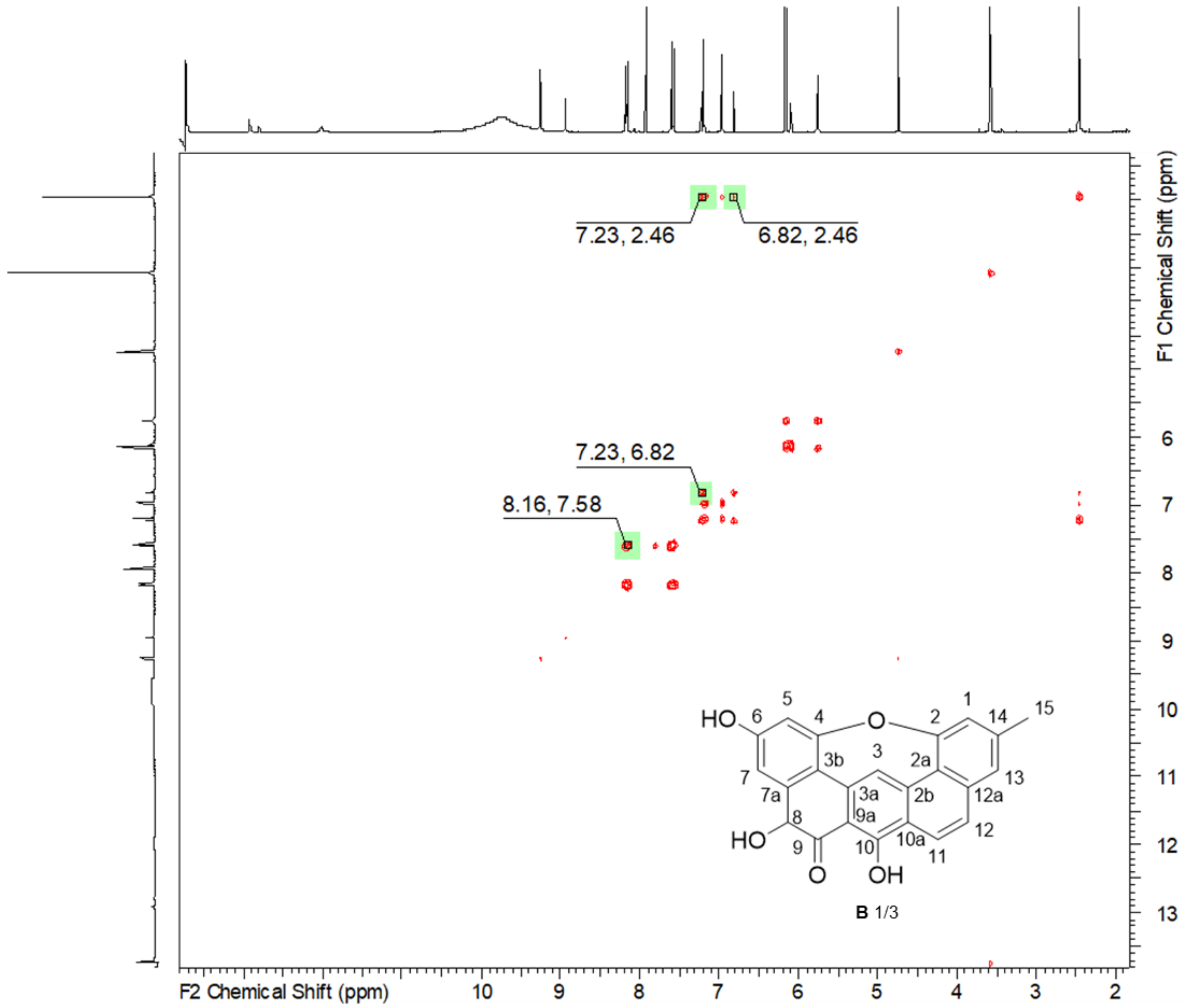

Figure S 31. COSY-spectrum of aggregicyclin in THF-d_8 at 700/175 MHz. Peaks marked were assigned to B derivative.

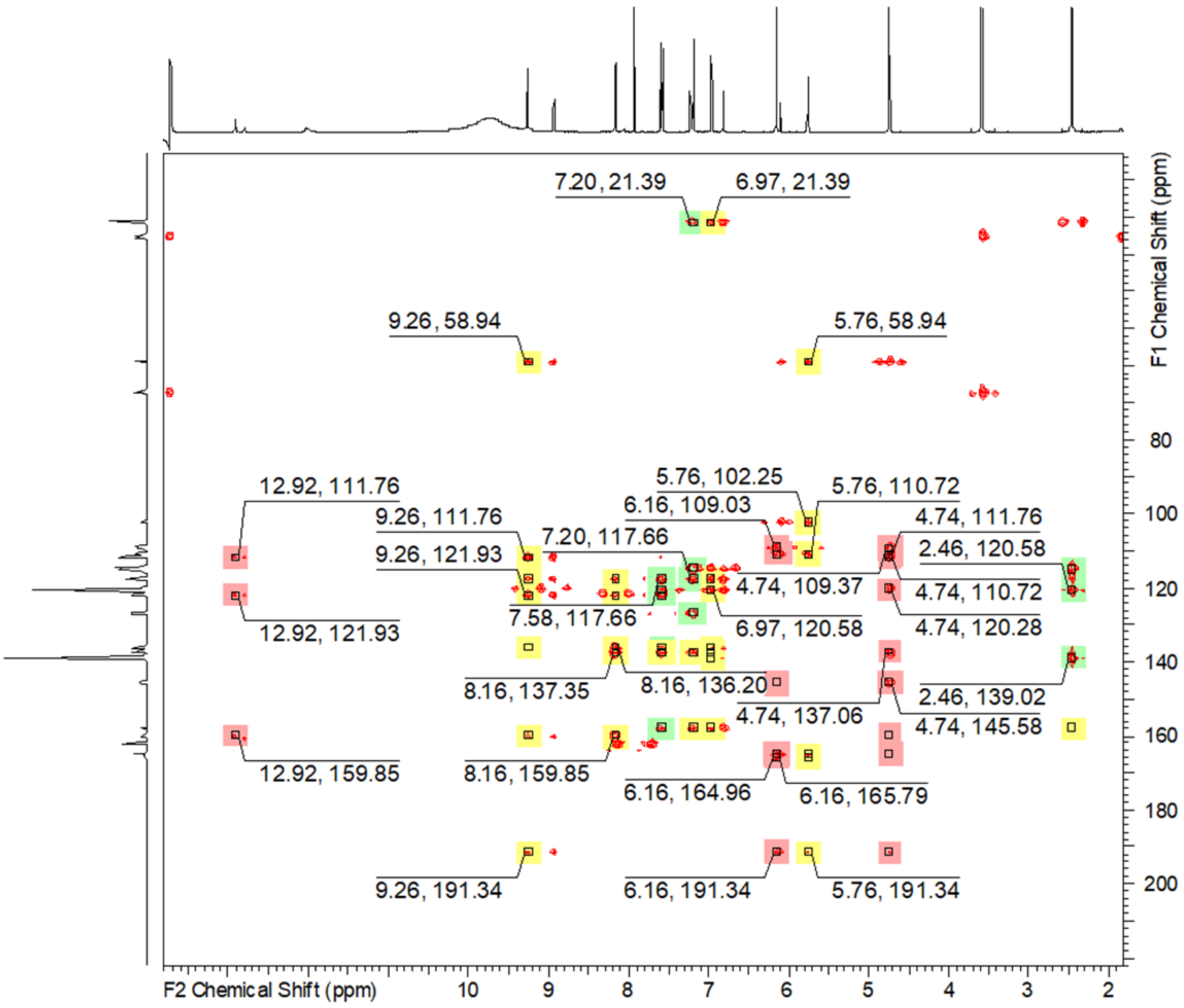

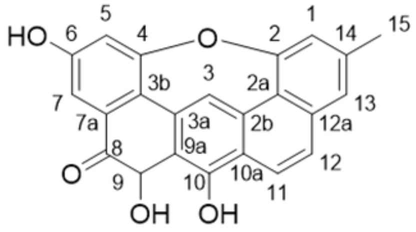

A 2/3

Figure S 32. HMBC-spectrum of aggregicyclin in THF-d_8 at 700/175 MHz optimized for constant 13 = 6 Hz. Peaks marked were assigned to A derivative.

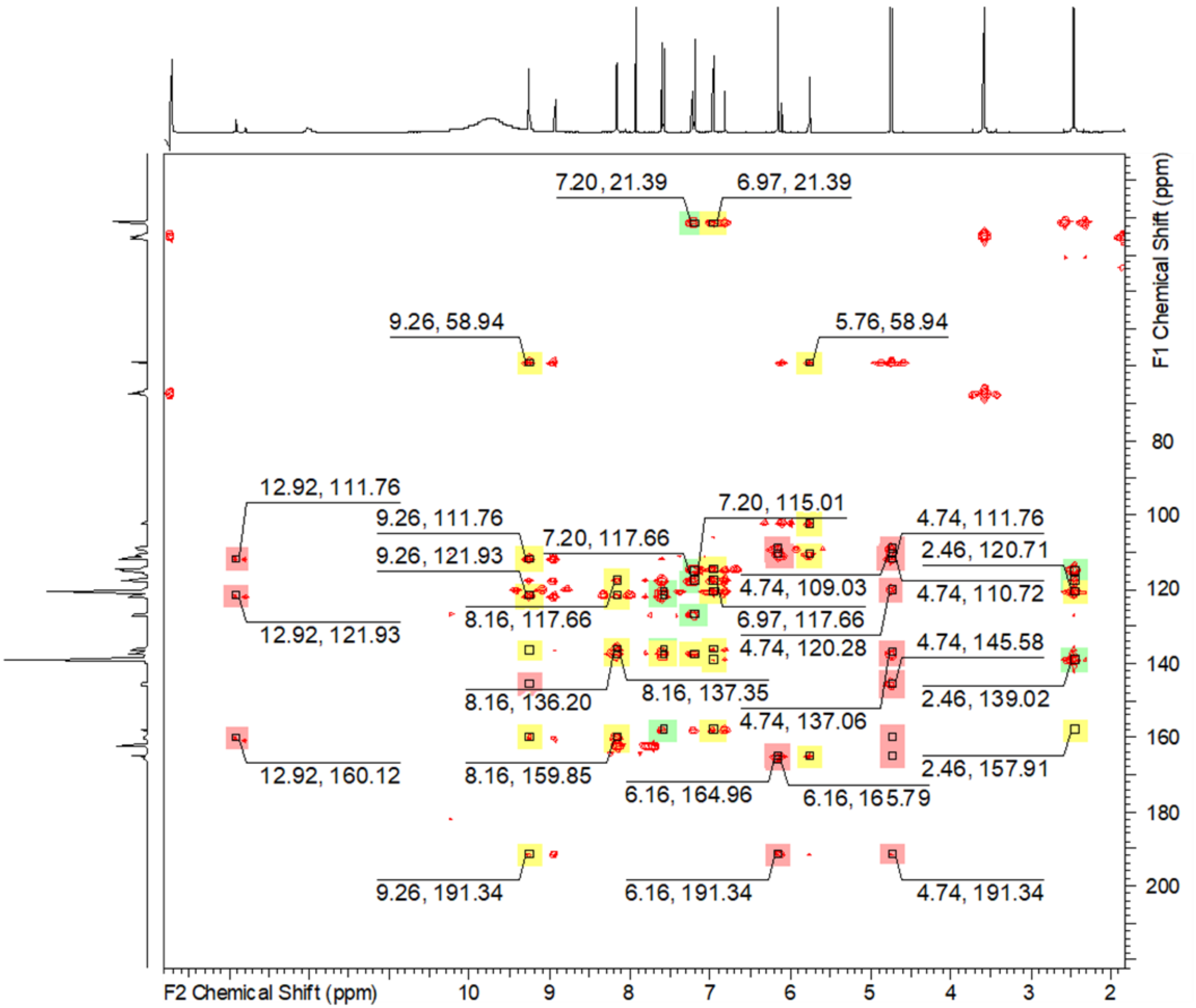

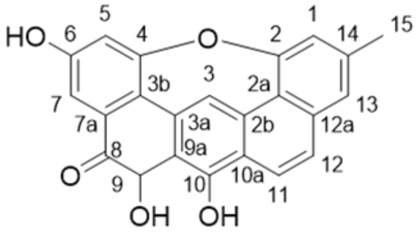

A 2/3

Figure S 33. HMBC-spectrum of aggregicyclin in THF-d8 at 700/175 MHz optimized for constant 13 = 10 Hz. Peaks marked were assigned to A derivative.

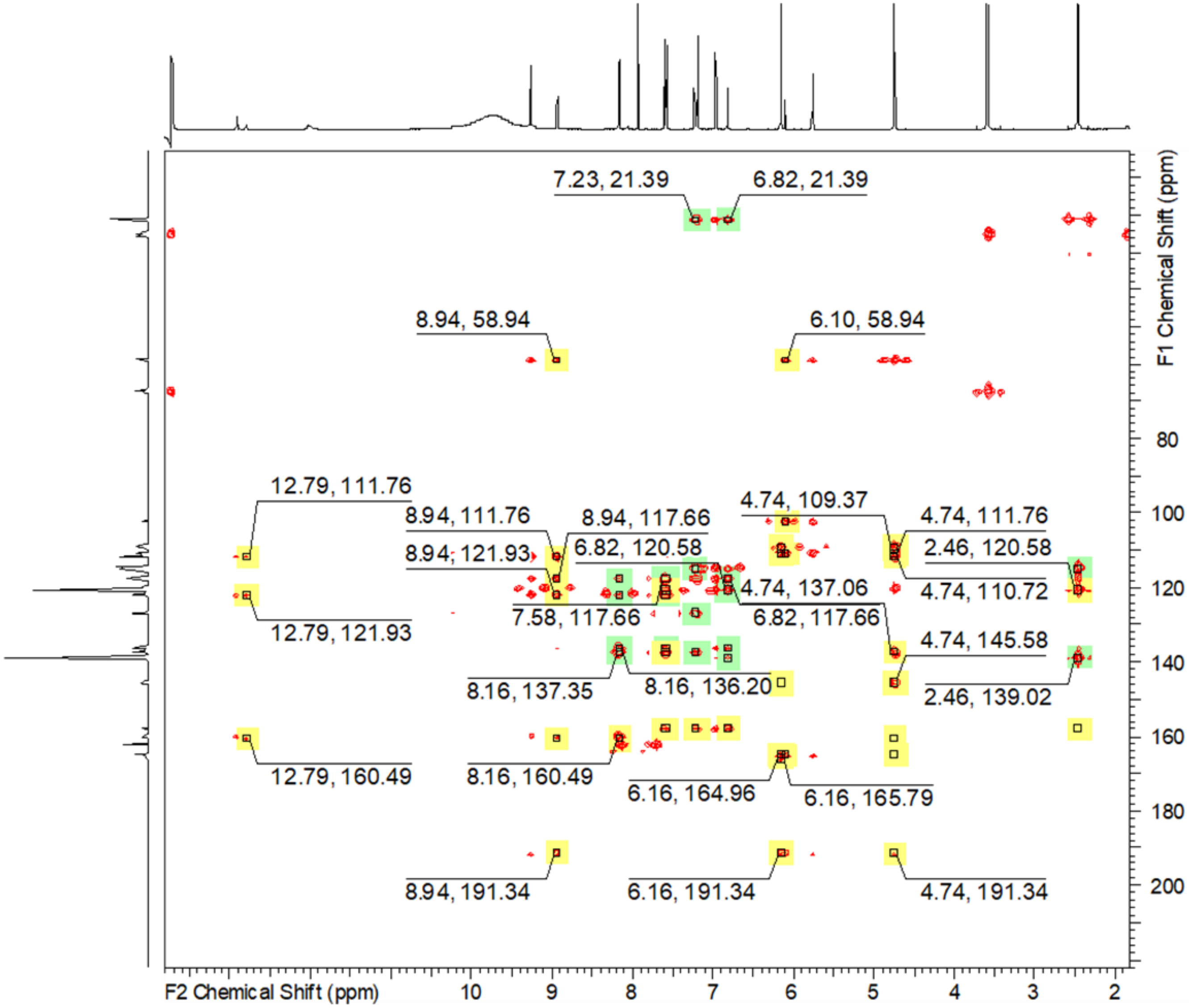

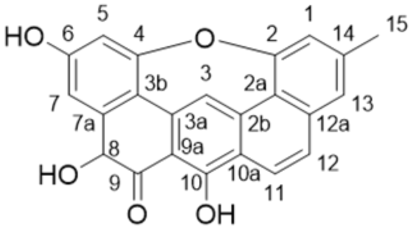

B 1/3

Figure S 34. HMBC-spectrum of aggregicyclin in THF-d8 at 700/175 MHz optimized for constant 13 = 6 Hz. Peaks marked were assigned to B derivative.

8 Biological assay conditions

Table S 10. Microbial test strains and positive controls used for MIC determination.

Microbial strain	Positive control
C. albicans	Amphotericin B
P. anomala	Amphotericin B
C. freundii	Ciprofloxacin-HCl
A. baumanii	Ciprofloxacin-HCl
S. aureus	Vancomycin
B. subtilis	Vancomycin
E. coli	Ciprofloxacin-HCl
P. aeruginosa	Ciprofloxacin-HCl
M. smegmatis	Rifampicin

Table S 11. MIC and IC_{50} values for Aggregicyclin

Microbial strain	MIC Aggregicyclin [μM]
C. albicans	>173
P. anomala	>173
C. freundii DSM 30039	>173
A. baumanii DSM 30008	>173
S. aureus Newman	22
B. subtilis DSM 10	86
E. coli BW25113	>173
P. aeruginosa PA14	>173
M. smegmatis mc² 155	n.d.
**Cancer cell line**	**IC_{50} Aggregicyclin [μM]**
HepG2	1.2
KB3.1	11
HCT116	1.8

9 References

(1) Sood, S.; Awal, R. P.; Wink, J.; Mohr, K. I.; Rohde, M.; Stadler, M.; Kämpfer, P.; Glaeser, S. P.; Schumann, P.; Garcia, R.; Müller, R. *Aggregicoccus edonensis* gen. nov., sp. nov., an unusually aggregating myxobacterium isolated from a soil sample. *Int J Syst Evol Microbiol* **2015**, *65* (Pt 3), 745–753. DOI: 10.1099/ijs.0.061176-0.

(2) Panter, F.; Krug, D.; Müller, R. Novel Methoxymethacrylate Natural Products Uncovered by Statistics-Based Mining of the *Myxococcus fulvus* Secondary Metabolome. *ACS Chem. Biol.* **2019**, *14* (1), 88–98. DOI: 10.1021/acschembio.8b00948.

(3) O'Leary, N. A.; Wright, M. W.; Brister, J. R.; Ciufo, S.; Haddad, D.; McVeigh, R.; Rajput, B.; Robbertse, B.; Smith-White, B.; Ako-Adjei, D.; Astashyn, A.; Badretdin, A.; Bao, Y.; Blinkova, O.; Brover, V.; Chetvernin, V.; Choi, J.; Cox, E.; Ermolaeva, O.; Farrell, C. M.; Goldfarb, T.; Gupta, T.; Haft, D.; Hatcher, E.; Hlavina, W.; Joardar, V. S.; Kodali, V. K.; Li, W.; Maglott, D.; Masterson, P.; McGarvey, K. M.; Murphy, M. R.; O'Neill, K.; Pujar, S.; Rangwala, S. H.; Rausch, D.; Riddick, L. D.; Schoch, C.; Shkeda, A.; Storz, S. S.; Sun, H.; Thibaud-Nissen, F.; Tolstoy, I.; Tully, R. E.; Vatsan, A. R.; Wallin, C.; Webb, D.; Wu, W.; Landrum, M. J.; Kimchi, A.; Tatusova, T.; DiCuccio, M.; Kitts, P.; Murphy, T. D.; Pruitt, K. D. Reference sequence (RefSeq) database at NCBI: current status, taxonomic expansion, and functional annotation. *Nucleic Acids Res.* **2016**, *44* (D1), D733-45. DOI: 10.1093/nar/gkv1189.

(4) Panter, F.; Krug, D.; Baumann, S.; Müller, R. Self-resistance guided genome mining uncovers new topoisomerase inhibitors from myxobacteria. *Chem. Sci.* **2018**, *9* (21), 4898–4908. DOI: 10.1039/C8SC01325J.

(5) Iniesta, A. A.; García-Heras, F.; Abellón-Ruiz, J.; Gallego-García, A.; Elías-Arnanz, M. Two systems for conditional gene expression in *Myxococcus xanthus* inducible by isopropyl-ß-D-thiogalactopyranoside or vanillate. *J. Bacteriol.* **2012**, *194* (21), 5875–5885. DOI: 10.1128/JB.01110-12.

(6) Magrini, V.; Storms, M. L.; Youderian, P. Site-specific recombination of temperate Myxococcus xanthus phage Mx8: regulation of integrase activity by reversible, covalent modification. *J. Bacteriol.* **1999**, *181* (13), 4062–4070. DOI: 10.1128/JB.181.13.4062-4070.1999.

(7) Orndorff, P.; Stellwag, E.; Starich, T.; Dworkin, M.; Zissler, J. Genetic and physical characterization of lysogeny by bacteriophage MX8 in *Myxococcus xanthus*. *J. Bacteriol.* **1983**, *154* (2), 772–779.

(8) Hug, J. J.; Panter, F.; Krug, D.; Müller, R. Genome mining reveals uncommon alkylpyrones as type III PKS products from myxobacteria. *J. Ind. Microbiol. Biotechnol.* **2019**, *46* (3-4), 319–334. DOI: 10.1007/s10295-018-2105-6.

(9) Lukežič, T.; Fayad, A. A.; Bader, C.; Harmrolfs, K.; Bartuli, J.; Groß, S.; Lešnik, U.; Hennessen, F.; Herrmann, J.; Pikl, Š.; Petković, H.; Müller, R. Engineering Atypical Tetracycline Formation in Amycolatopsis sulphurea for the Production of Modified Chelocardin Antibiotics. *ACS Chem. Biol.* **2019**, *14* (3), 468–477. DOI: 10.1021/acschembio.8b01125.

(10) Araya-Maturana, R.; Pessoa-Mahana, H.; Weiss-López, B. Very Long-Range Correlations (nJC,H n > 3) in HMBC Spectra. *Nat. Prod. Commun.* **2008**, *3* (3), 1934578X0800300. DOI: 10.1177/1934578X0800300321.

(11) Nicoud, R.-M.; Jaubert, J.-N.; Rupprecht, I.; Kinkel, J. Enantiomeric enrichment of non-racemic mixtures of binaphthol with non-chiral packings. *Chirality* **1996**, *8* (3), 234–243. DOI: 10.1002/(SICI)1520-636X(1996)8:3<234:AID-CHIR2>3.0.CO;2-H.
